# Impact of Pharmacovigilance Interventions Targeting Fluoroquinolones on Antibiotic Use in the Netherlands and the United Kingdom

**DOI:** 10.1002/pds.70081

**Published:** 2025-01-16

**Authors:** Tomas Lasys, Yared Santa‐Ana‐Tellez, Satu J. Siiskonen, Rolf H. H. Groenwold, Helga Gardarsdottir

**Affiliations:** ^1^ Division of Pharmacoepidemiology and Clinical Pharmacology, Utrecht Institute for Pharmaceutical Sciences (UIPS) Utrecht University Utrecht The Netherlands; ^2^ Department of Clinical Epidemiology Leiden University Medical Centre Leiden The Netherlands; ^3^ Department of Pharmaceutical Sciences, School of Health Sciences University of Iceland Reykjavik Iceland; ^4^ Department of Clinical Pharmacy University Medical Centre Utrecht Utrecht The Netherlands

**Keywords:** antibiotics, fluoroquinolones, impact studies, pharmacovigilance, risk minimization measures

## Abstract

**Purpose:**

Fluoroquinolones are antibiotics associated with adverse events that prompted the European Medicines Agency to implement risk minimization measures (RMMs) in 2018/19 and 2020. Our aim is to assess the RMMs' impact on antibiotic prescriptions in primary care during 2014–2023.

**Methods:**

We assessed antibiotic prescriptions using CPRD GOLD (the United Kingdom, UK) and PHARMO (the Netherlands, NL). Prescriptions were assessed for fluoroquinolones and alternative antibiotics. The impact of RMMs on prescribing was assessed with interrupted time series (ITS) using monthly prescription rates per 10 000 person‐years (MPTPY).

**Results:**

Between 2014 and 2023, we identified cohorts of 4.0 (UK) and 0.9 million (NL) antibiotic users. Fluoroquinolones were prescribed to initiate 1.5% (UK) to 5.8% (NL) of the treatment episodes.

Fluoroquinolone prescribing before the RMMs slowly decreased in the UK and was stable in the NL. The 2018/19 RMMs were associated with a steady downward post‐RMMs trend in incident use of fluoroquinolones (MPTPY −0.7 [UK] and −0.8 [NL]) and opposite changes after 2020 RMMs (MPTPY 0.6 [UK] and 1.8 [NL]).

The 2018/2019 RMMs were linked with increasing trends for other antibacterials (J01XX) in both countries and other beta‐lactam antibacterials in the UK, but most antibiotics had decreasing trends post‐RMMs in both countries. After the 2020 RMMs, some antibiotic groups showed upward trends.

**Conclusion:**

The risk minimization measures in 2018/2019 were associated with a moderate decrease in fluoroquinolone prescribing, with no further decrease after 2020 RMMs. There was no sustained increase in other antibiotic prescribing, suggesting that overprescribing was negligible as an unintended impact of RMMs.

**Summary**

- The 2018/19 risk minimization measures (RMMs) targeting fluoroquinolones showed a moderate impact in reducing fluoroquinolone prescribing in both countries.
- No further reduction in fluoroquinolone prescribing was seen after implementing 2020 RMMs.
- Similar use patterns were seen irrespective of prescribing type (incident, add‐on, and continued use).
- An increasing trend of some other antibiotics was observed following the 2018/19 RMMs. However, most other antibiotics had decreasing trends and do not suggest a noteworthy unintended impact of the RMMs.
- It remains unclear whether changes in antibiotic prescribing reflect added health benefits for patients by avoiding harmful adverse events.

## 1 Introduction

(Fluoro)quinolones are a class of synthetic broad‐spectrum antibiotics. Their use has been associated with the risk of serious adverse events, including electrocardiographic abnormalities and tendon and joint disorders [1, 2]. Regulatory authorities in European countries imposed restrictions on moxifloxacin prescribing in 2007–2009, ciprofloxacin in 2008, and levofloxacin in 2012 due to safety concerns [3]. In 2017, the European Medicines Agency (EMA) started a new safety assessment procedure that concluded that severe adverse reactions to (fluoro)quinolones could be long‐lasting, disabling and potentially irreversible [4]. These reactions, including tendon, muscle and joint disorders, and neurologic and psychiatric disorders, can disrupt a patient's daily activities.

Various risk minimization measures (RMMs) were implemented, including revised indications, safety warnings, Direct Healthcare Professional Communication (DHPC), and market withdrawal of quinolones. A DHPC disseminated in 2019 recommended against prescribing fluoroquinolones for milder, nonsevere, or self‐limiting infections and other indications for which they are not the first line of treatment [5]. In 2020, the EMA issued another EU‐wide DHPC warning against fluoroquinolone use in patients with certain health conditions because of the higher risk of heart valve regurgitation or heart valve incompetence [6].

These regulatory interventions aimed to reduce severe and/or long‐lasting adverse events associated with fluoroquinolone usage. While several studies assessed their impact on fluoroquinolone use patterns, the impact was not assessed in relation to the prescribing of alternative antibiotics [7, 8, 9, 10]. Also, the potential unintended impact of increased use of alternative antibiotics has not been assessed in detail. In this case, switching to other antibiotics could cause suboptimal initial treatment, prolonged treatment duration, increased risk of antimicrobial resistance and increased consumption of reserve antibiotics [11].

This study aimed to assess the impact of regulatory interventions targeting fluoroquinolones on antibiotic use patterns in primary care. As a possible unintended impact of RMMs, we assessed prescribing patterns of other antibiotics.

## 2 Methods

### 2.1 Setting and Data Sources

We analyzed data from two primary care data sources: the Clinical Practice Research Datalink (CPRD) GOLD from the United Kingdom (UK) and the PHARMO Data Network from the Netherlands (NL).

The CPRD GOLD receives de‐identified health records routinely collected from primary care practices in the UK, encompassing medical conditions, prescriptions, and referrals, with potential links to other databases, and it covers approximately 18 million patients [12, 13, 14]. The research protocol was approved by the CPRD's Research Data Governance Process for Medicines and Healthcare Products Regulatory Agency Database Research (protocol 23_002550).

The PHARMO Data Network comprises dispensing data that can be linked to other primary and secondary healthcare data at a patient level [15, 16]. The PHARMO Data Network collects information from around 2000 primary and secondary care practices covering approximately 10 million people in the Netherlands. This study included only subjects enrolled in dispensing and general practice (GP) databases.

### 2.2 Study Population

The study cohort included patients enrolled in the CPRD or PHARMO with a prescription (in CPRD) or dispensing (in PHARMO) of any antibiotic for systemic use (ATC codes J01* and BNF codes 501*) between January 1, 2014 and December 31, 2021 (in PHARMO) or October 31, 2023 (in CPRD). In the text, we refer to prescriptions (where, for the PHARMO database, this can be read as dispensing). Monthly prescribing rates were estimated using the entire database population.

Patients were included at the date of the first incident prescription of antibiotics (index date) after they had been enlisted in the database for at least 365 days. Follow‐up lasted until death, deregistration, or study end, whichever came first (see Supporting Information: Section S1.2).

### 2.3 Outcomes

Treatment episodes were constructed with an estimated duration of each prescription defined from the starting date of the prescription to the estimated end date, which was calculated by dividing the quantity of medicine prescribed by the treatment regimen. If information on the treatment regimen was unavailable, the standard defined daily dose (DDD) was used [17]. Subsequent antibiotic prescriptions within a 30‐day permissible gap after the estimated end date of the prior prescription were considered part of the same treatment episode. An overlapping period of prescriptions of the same product was appended to the end of the last prescription (see Supporting Information: Section S1.3). A subsequent prescriptions prescribed following a gap larger than 30 days was consider incident use.

Prescriptions were categorized as incident, add‐on, or continued use. The first prescription in the treatment episode was considered incident use. Subsequent prescriptions were classified as treatment continuation (if the same antibiotic was prescribed) or treatment add‐on (if the subsequent prescription during the same treatment episode was for a different antibiotic). The term add‐on was used because, due to short expected treatment durations for antibiotics (usually requiring one prescription) and lack of diagnosis, verifying whether another antibiotic was used as a switch for the same indication was not possible.

The study's primary outcome was the impact of RMMs on prescribing rates of systemic antibiotics (ATC J01). Prescribing rates were assessed as the monthly number of prescriptions per 10 000 person‐years (MPTPY) contributing to the database that month. Prescribing rates were assessed based on prescription category (incident, add‐on, or continued use).

**FIGURE 1:**
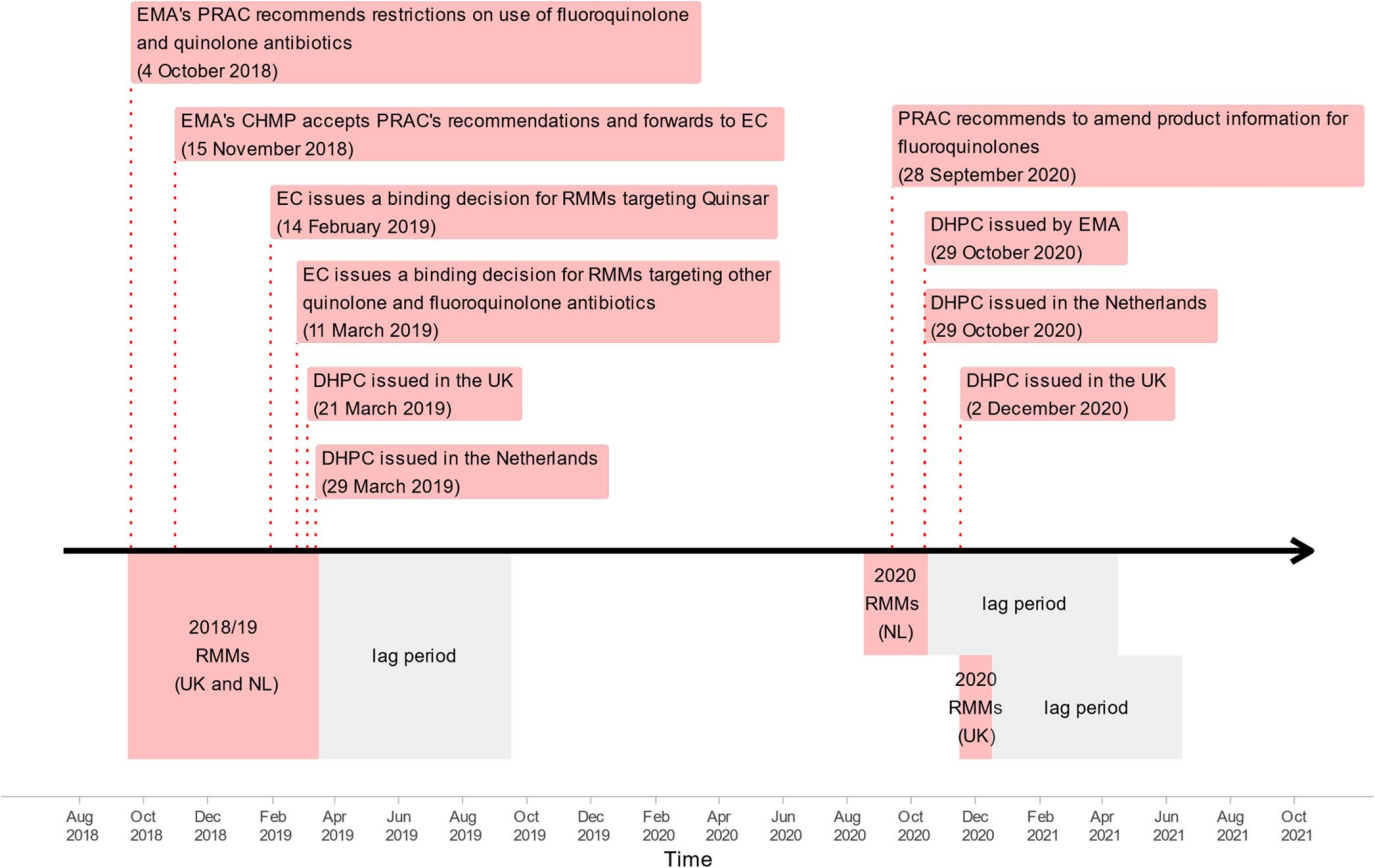
Timeline of risk minimization measures and related events targeting fluoroquinolones. The graph depicts a timeline of individual risk minimization measures (RMMs) and related events used to construct RMMs period definitions. Red areas at the bottom show RMMs period definitions, and gray areas show the applied lag periods used in the main interrupted time series analysis. Both countries' 2018/19 RMMs period was from October 2018 to March 2019 (lag period until September 2019). Since the UK withdrew from the European Union after February 2020, the second RMM periods are presented separately for each country: From September 2020 to October 2020 for NL (lag period until May 2021) and December 2020 for the UK (lag period until June 2021). Dates and RMMs periods in tabular form are presented in the Supporting Information: Section S1.1. Only data from complete calendar months will be included in the analysis. Months that are partially covered by intervention or lag periods will be excluded. EC: European Commission, EMA: European Medicines Agency, DHPC: Direct Healthcare Professional Communication, PRAC: Pharmacovigilance Risk Assessment Committee, NL: The Netherlands, RMMs: Risk minimization measures, UK: United Kingdom.

Antibiotic prescribing patterns were studied from January 1, 2014 until December 31, 2021 (PHARMO) or October 31, 2023 (CPRD). During that period, two RMMs periods were defined for each country based on regulatory events: the first from October 2018 until March 2019 was the same for both countries (referred to as 2018/19 RMMs), and the second from September until October in 2020 for the Netherlands and until December 2020 for the UK (referred as 2020 RMMs). The RMMs' summary is presented in Figure 1 and Supporting Information: Section S1.1.

### 2.4 Covariates

We collected covariate information at the start of each treatment episode. It included age, sex, indication for antibiotic use, and risk factors related to increased risk for tendonitis/tendon rupture and aortic aneurysm/aortic dissection. Antibiotic treatment indications were based on diagnoses typically treated with antibiotics and were determined in this order: (1) diagnosis on the prescription date, (2) closest diagnosis up to 7 days before, or (3) closest diagnosis within 7 days after the prescription. For codes used to identify covariates, see Supporting Information: Section S2.

All covariates were used in the stratified or subgroup analyses; covariate values at the index date were used to describe the cohort.

### 2.5 Data Analysis

Analyses for each database followed the same methodology, employing descriptive statistics for patients' characteristics at cohort entry and interrupted time‐series (ITS) analyses to evaluate the impact of RMMs on monthly prescription patterns for incident, add‐on, and continued prescriptions. Time series were modeled using Gaussian generalized linear models with three segments: estimating preintervention patterns, patterns between RMMs, and patterns after 2020 RMMs. Data during RMMs periods and 6 months after were excluded from the main model to mitigate lag effects [18]. A graphical depiction of RMMs and lag periods is presented in Figure 1. Estimated step and slope coefficients indicated immediate changes and changes in trends (see Supporting Information: Section S1.4).

We conducted additional analyses stratifying for age, sex, antibiotic indication, and subgroups at heightened risk for tendinitis or tendon rupture and aortic aneurysm or dissection. Additional sensitivity analyses were performed using different lag periods after RMMs (0–5 months), different permissible gap durations (7, 10, 14, and 30 days), and including seasonal trends. To check data robustness, we also constructed ITS Poisson regression models and models excluding data after the onset of the COVID‐19 pandemic (the first cases were reported on January 31, 2020 in the UK and February 27, 2020 in the Netherlands).

The absence of relevant information on indication was classified as an unknown indication, while the absence of information on risk factors was viewed as the absence of corresponding conditions. Patients with missing values for age and sex were omitted from the analysis.

A significance level of *α* = 0.05 was used in all statistical tests. Data were prepared and analyzed using SAS 9.3 and R version 4.2.3.

## 3 Results

### 3.1 Cohort Description

**FIGURE 2:**
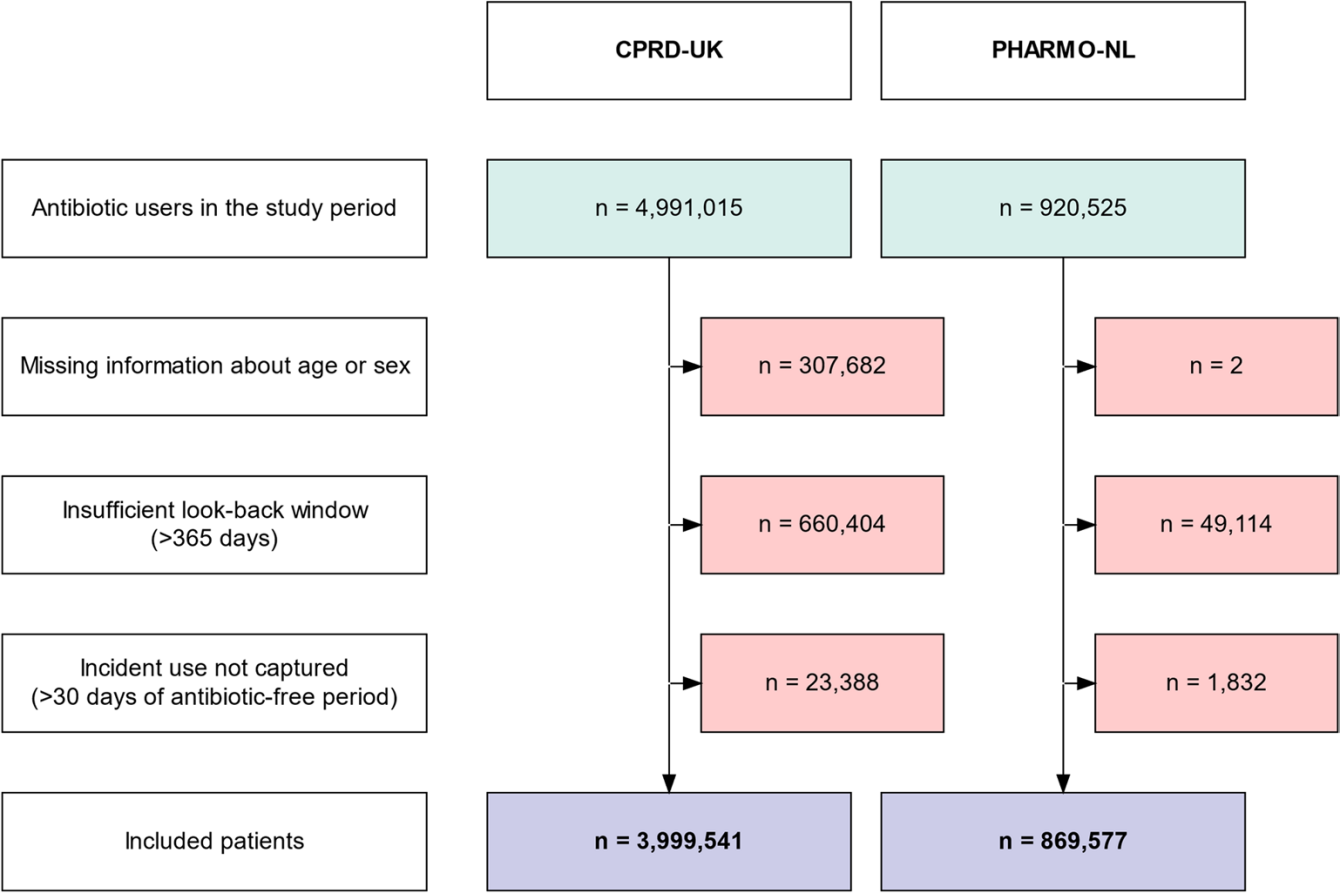
Flowchart. Study subject selection process. The source population included all people registered and validated in the CPRD GOLD database between January 2014 and October 2023. The source population included all patients registered (active) in the PHARMO outpatient pharmacy and registered (active) in the PHARMO GP data between January 2014 and December 2021.

We included 3 999 541 patients from CPRD (2014–2023) and 869 577 patients from PHARMO (2014–2021). Figure 2 shows the selection process.

In the UK cohort, 56.8% were female, with a median age of 42 (*Q*1 = 21, *Q*3 = 62) at the index date. In the Netherlands (NL) cohort, 56.8% were female, with a median age of 48 years (*Q*1 = 26, *Q*3 = 64) (Table 1).

**TABLE 1:** Patient characteristics at index date.

Variable	Description	CPRD‐UK	PHARMO‐NL
N of patients		3 999 541	869 577
Sex	Female	2 273 062 (56.8%)	493 847 (56.8%)
Age	Q1	21.0	26.0
	Median	42.0	48.0
	Mean	42.1	45.3
	Q3	62.0	64.0
Follow‐up (in months)	Q1	16.5	33.8
	Median	44.5	58.8
	Mean	51.2	56.6
	Q3	84.7	80.8
Risk group^a^, ^b^	Risk factors for aortic aneurysm or dissection	1 833 765 (45.8%)	257 010 (29.6%)
	Risk factors for tendonitis or tendon rupture	1 561 515 (39.0%)	115 637 (13.3%)
	No known risk factors	2 033 715 (50.8%)	573 389 (65.9%)

^a^ Details of individual risk factors used to construct risk groups are presented in Supporting Information: Section S1.5.

^b^ In the analysis, the presence of risk factors was assessed at the start of each treatment episode.

We identified 13 665 964 antibiotic treatment episodes for the UK cohort during the study period, 57.2% of these treatment episodes were started with any penicillin, and 1.6% started with fluoroquinolones. In the NL cohort, we identified 2 232 017 treatment episodes, 45.7% of these treatment episodes were initiated with penicillins, and 5.8% started with fluoroquinolones (see Table 2 and Supporting Information: Section S1.6).

**TABLE 2:** Summary of antibiotic treatment episodes.

Variable	Description	CPRD‐UK	PHARMO‐NL
N of treatment episodes		13 665 964	2 232 017
N of prescriptions		20 602 848	3 127 054
First prescribed antibiotic during treatment episode^a^	Penicillins with extended‐spectrum	4 322 492 (31.6%)	540 212 (24.2%)
	Beta‐lactamase resistant penicillins	1 972 608 (14.4%)	169 773 (7.6%)
	Tetracyclines	1 740 149 (12.7%)	312 661 (14.0%)
	Sulfonamides and trimethoprim	1 532 790 (11.2%)	51 493 (2.3%)
	Macrolides	1 234 103 (9.0%)	198 929 (8.9%)
	Beta‐lactamase sensitive penicillins	1 055 631 (7.7%)	49 360 (2.2%)
	Nitrofuran derivatives	1 015 936 (7.4%)	433 547 (19.4%)
	Combinations of penicillins	473 682 (3.5%)	261 449 (11.7%)
	Fluoroquinolones	215 476 (1.6%)	128 393 (5.8%)
	Other antibiotics	275 702 (2.0%)	108 656 (4.7%)
Diagnosis at the start of treatment episode^a^	Urinary tract infection	366 678 (2.7%)	152 837 (6.8%)
	Upper respiratory tract infection	570 123 (4.2%)	141 016 (6.3%)
	Skin and soft tissue infection	187 462 (1.4%)	94 573 (4.2%)
	Lower respiratory tract infection	891 501 (6.5%)	93 912 (4.2%)
	Other	645 887 (4.7%)	103 206 (4.6%)
	Unknown	11 021 576 (80.6%)	1 657 273 (74.3%)
Single prescription during the treatment episode		10 425 695 (76.3%)	1 815 253 (81.3%)
N of prescriptions per treatment episode	Q1	1.0	1
	Median	1.0	1
	Mean	1.5	1.4
	Q3	1.0	1
N of different products per treatment episode	Q1	1.0	1
	Median	1.0	1
	Mean	1.2	1.2
	Q3	1.0	1

*Note:* If the treatment episode was initiated with more than one drug simultaneously, each drug was counted as the first one.

^a^ If the start of the treatment episode was associated with more than one diagnosis, each diagnosis was counted as the first.

Other quinolones were rarely used to start treatment, with only 3 cases in the UK and 94 cases in the Netherlands, with the last prescriptions recorded in 2015 (UK) and 2016 (NL).

### 3.2 Fluoroquinolone Prescribing

**FIGURE 3:**
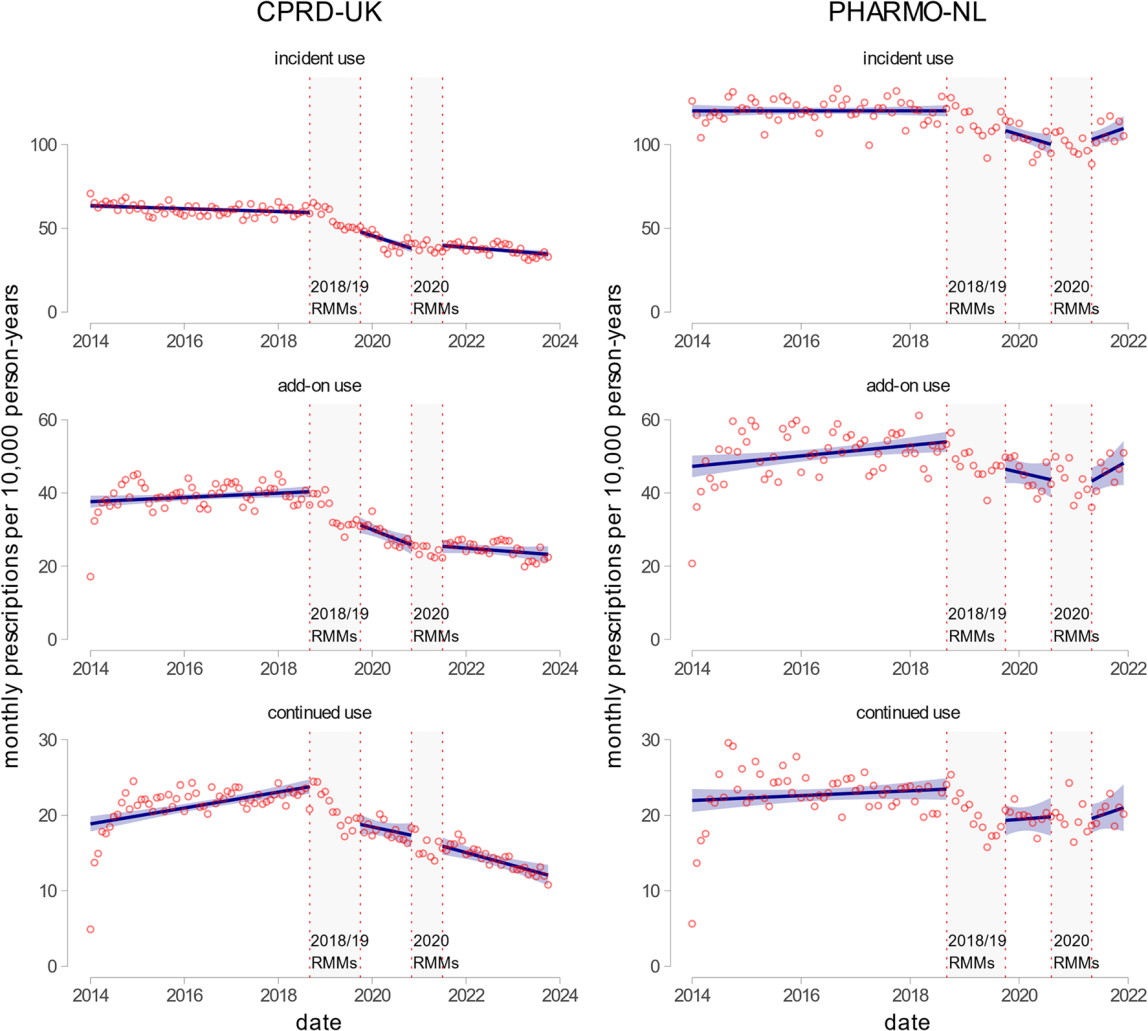
Prescription rates of fluoroquinolones depending on the use category (from top to bottom: incident use, add‐on use, and continued use). The left panel shows fluoroquinolone prescribing rates in CPRD‐UK data, and the right panel shows fluoroquinolone prescribing rates in PHARMO‐NL data. Points in the graphs indicate measured monthly prescriptions, while lines indicate estimated prescribing rates based on interrupted time series (ITS) models. Rates are measured as monthly prescriptions per 10 000 person‐years (MPTPY). Vertical lines indicate risk minimization measures and their lag periods used in the analysis: 2018/19 RMMs—risk minimization measures from October 2018 until March 2019 (for both countries) with a 6‐month lag period (until September 2019). 2020 RMMs—risk minimization measures from September 2020 until October 2020 (for the Netherlands) and only December 2020 (for the UK) with 6‐month lag periods (May and June 2021, respectively).

In the UK, the estimated prescribing of fluoroquinolones in January 2014 was 63.5 (95% CI 61.9 to 65.1) monthly prescriptions per 10 000 person‐years (MPTPY), with ciprofloxacin making up 92.0% of all fluoroquinolone prescriptions. Before the implementation of the RMMs, the treatment initiation with fluoroquinolones had a slowly decreasing trend of −0.1 (95% CI −0.1 to −0.002) MPTPY. Following the 2018/19 RMMs, a downward trend (−0.7 [95% CI −1.0 to −0.4] MPTPY) in prescribing was observed. However, the second RMMs were followed by increased prescribing of fluoroquinolones (0.6 [95% CI 0.3 to 0.9] MPTPY). In the Netherlands, the estimated use of fluoroquinolones in January 2014 was 120.1 (95% CI 116.4 to 123.8) MPTPY. Ciprofloxacin was the most prescribed fluoroquinolone, accounting for 82.0% of all prescriptions. Before the RMMs, the treatment initiation with fluoroquinolones was stable (95% CI −0.1 to 0.1) MPTPY. After the 2018/19 RMMs, a decreasing trend of prescribing was observed (−0.8 [95% CI −1.6 to −0.0] MPTPY). The 2020 RMMs were associated with increasing trend (1.8 [95% CI 0.4 to 3.1]). See Figure 3 and Table 3.

**TABLE 3:** Interrupted time series regression analysis for Fluoroquinolones prescription rates depending on the category of use (incident use, add‐on use, and continued use) in 2014–2021 (PHARMO‐NL) and 2014–2023 (CPRD‐UK).

Database	Type of use	(Intercept) [95% CI]	Slope before RMMs [95% CI]	Step change after 2018/19 RMMs [95% CI]	Slope change after 2018/19 RMMs [95% CI]	Step change after 2020 RMMs [95% CI]	Slope change after 2020 RMMs [95% CI]
CPRD‐UK	Incident use	**63.470** [**61.860 to 65.081**] ***p*** < **0.001**	−**0.072 [**−**0.118 to** −**0.026] *p*** = **0.003**	−**5.887** [−**9.855 to** −**1.920] *p*** = **0.004**	−**0.680** [−**0.950 to** −**0.410**] ***p*** < **0.001**	3.719 [0.014 to 7.425] *p* = 0.052	**0.564** [**0.272 to 0.856**] ***p*** < **0.001**
	Add‐on use	**37.556** [**35.870 to 39.242**] ***p*** < **0.001**	**0.049** [**0.001 to 0.097**] ***p*** = **0.049**	−**6.602** [−**10.755 to** −**2.448**] ***p*** = **0.002**	−**0.455** [−**0.738 to** −**0.172] *p*** = **0.002**	0.526 [−3.353 to 4.405] *p* = 0.791	**0.325** [**0.020 to 0.631**] ***p*** = **0.039**
	Continued use	**18.771** [**17.739 to 19.803**] ***p*** < **0.001**	**0.088** [**0.059 to 0.117**] ***p*** < **0.001**	−**4.746** [−**7.288 to** −**2.204**] ***p*** < **0.001**	−**0.198** [−**0.371 to** −**0.025**] ***p*** = **0.027**	−0.340 [−2.714 to 2.034] *p* = 0.780	−0.032 [−0.219 to 0.155] *p* = 0.736
PHARMO‐NL	Incident use	**120.136** [**116.423 to 123.849**] ***p*** < **0.001**	0.001 [−0.104 to 0.107] *p* = 0.983	−6.048 [−16.002 to 3.906] *p* = 0.237	−**0.826** [−**1.606 to** −**0.047**] ***p*** = **0.041**	−2.183 [−14.644 to 10.278] *p* = 0.732	**1.778** [**0.448 to 3.108**] ***p*** = **0.011**
	Add‐on use	**47.154** [**44.118 to 50.190**] ***p*** < **0.001**	**0.120** [**0.034 to 0.206**] ***p*** = **0.008**	−6.191 [−14.330 to 1.947] *p* = 0.140	−0.409 [−1.046 to 0.229] *p* = 0.213	−4.817 [−15.006 to 5.371] *p* = 0.357	1.000 [−0.088 to 2.087] *p* = 0.075
	Continued use	**21.935** [**20.372 to 23.499**] ***p*** < **0.001**	0.027 [−0.018 to 0.071] *p* = 0.240	**−4.652** [−**8.844 to** −**0.461] *p*** = **0.033**	0.022 [−0.307 to 0.350] *p* = 0.897	−1.771 [−7.018 to 3.476] *p* = 0.510	0.157 [−0.403 to 0.717] *p* = 0.585

*Note:* 2018/19 RMMs—risk minimization measures and related regulatory events from October 2018 until March 2019 (for both countries). 2020 RMMs—risk minimization measures and related regulatory events September 2020 until October 2020 for NL and December 2020 for the UK. Model parameters are estimated for monthly prescription rates per 10 000 person‐years (MPTPY). In addition to RMMs, a 6‐month lag period is excluded from the model estimation. Bold values represent statistical significance at the *p* < 0.05.

Abbreviation: CI: confidence intervals.

In the UK, fluoroquinolone prescribed for add‐on and continued use had similar patterns to incident use but at lower rates. In contrast, trends for add‐on use did not demonstrate any impact of RMMs in the NL. The proportions of fluoroquinolone prescriptions by use type remained relatively stable throughout the study period (incident use was ~62% in the Netherlands and ~50% in the UK).

Fluoroquinolone use trends are presented in Figure 3 and Table 3. The data of individual fluoroquinolones are presented in Supporting Information: Section S1.7.

### 3.3 Prescribing of Other Antibiotics

**FIGURE 4:**
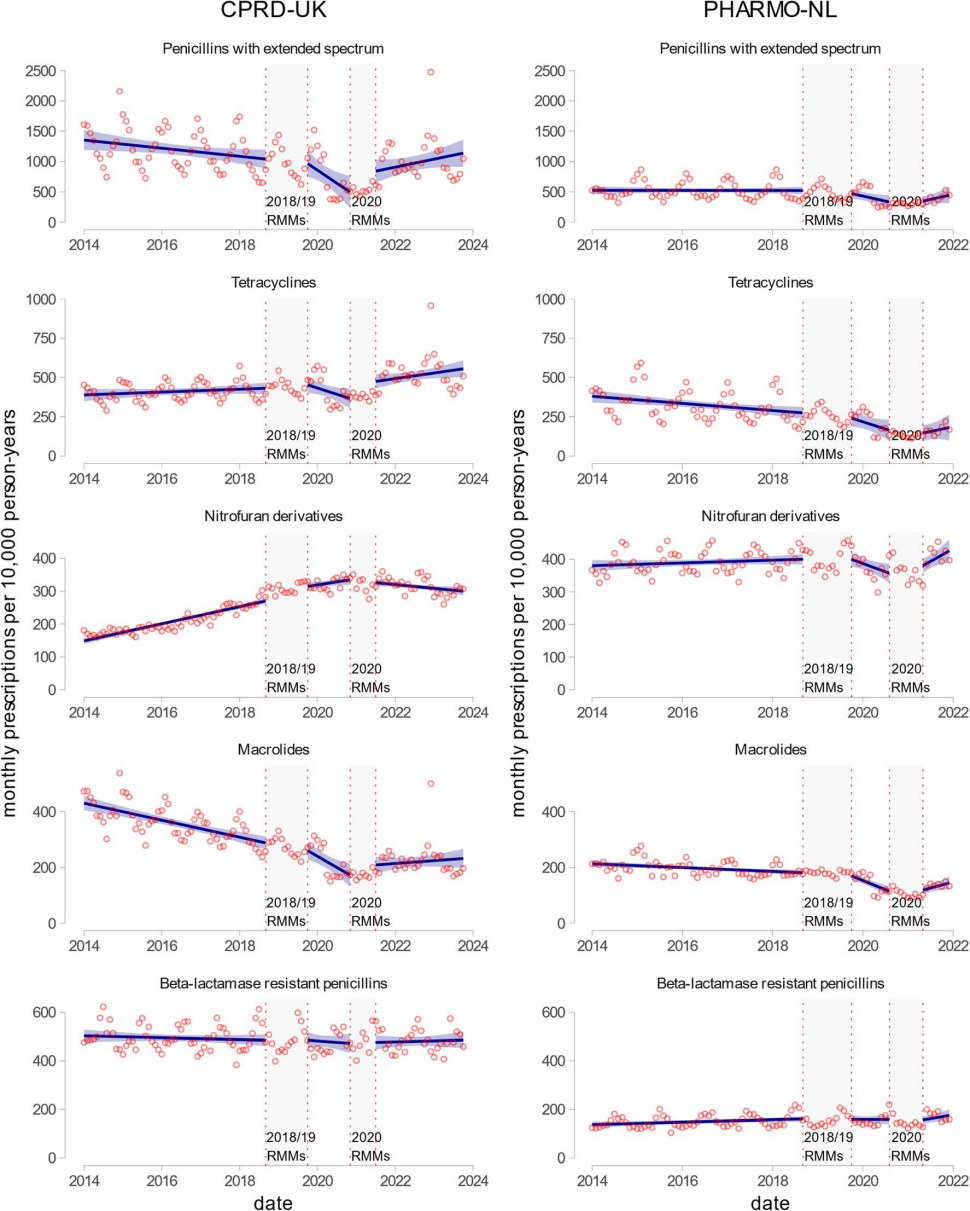
Prescription rates of 5 the most prescribed systemic antibiotic groups for incident use (based on 5th level ATC code) in the Netherlands and the UK. The left panel shows trends of fluoroquinolone use in CPRD‐UK data (the UK), and the right panel shows fluoroquinolone prescribing trends in PHARMO‐NL data (the Netherlands). Points in the graphs indicate measured monthly prescriptions, while lines indicate estimated prescribing rates based on interrupted time series (ITS) models. Rates are measured as monthly prescriptions per 10 000 person‐years (MPTPY). Vertical lines indicate risk minimization measures and their lag periods used in the analysis: 2018/19 RMMs—risk minimization measures from October 2018 until March 2019 (for both countries) with a 6‐month lag period (until September 2019). 2020 RMMs—risk minimization measures from September 2020 until October 2020 (for the Netherlands) and only December 2020 (for the UK) with 6‐month lag periods (May and June 2021, respectively). The high prescribing rates of penicillins with extended‐spectrum, tetracyclines, and macrolides in December 2022 are related to the surge in type A streptococcus infections during that month [19].

In the UK, most antibiotics had stable or decreasing trends before RMMs, while in the Netherlands, the antibiotic trends were mainly increasing. In the UK, the 2018/19 RMMs were associated with the increasing trend of other antibacterials (J01XX) (0.4 [95% CI 0.3 to 0.4]) and sulfonamides and trimethoprim (2.4 [95% CI 0.2 to 4.5]) and other beta‐lactam antibacterials (0.8 [95% CI 0.4 to 1.1]). However, some groups had decreasing trends after 2018/19 RMMs, notably penicillins with extended‐spectrum (−30.3 [95% CI 58.7 to −1.9] MPTPY) and tetracyclines (−7.4 [95% CI −14.2 to −0.6] MPTPY). In the Netherlands, there was an upward shift in prescribing trends after the 2018/19 RMMs only for other antibacterials (J01XX) (0.8 [95% CI 0.1 to 1.5]) while multiple antibiotic groups had decreasing trends, including penicillins with extended‐spectrum (11.9 [95% CI −22.0 to −1.8] MPTPY), tetracyclines (−6.2 [95% CI −12.1 to −0.3] MPTPY), and macrolides (−4.7 [95% CI −6.4 to −2.9] MPTPY). The 2020 RMMs were associated with increasing prescribing trends for most antibiotics in both countries (see Figure 4 and Supporting Information: Section S1.8).

### 3.4 Covariates

Throughout the study period, fluoroquinolones were prescribed more often in age groups over 60 years, more often for men. The leading indication for fluoroquinolones was urinary tract infections in both countries. However, no relevant diagnosis was available for 84.9% of the prescribing of antibiotics in the UK and 77.4% in the Netherlands. In the UK, in 67.9% of treatment episodes where fluoroquinolones were used, patients had conditions increasing cardiovascular risk, and 55.3% were at risk for tendon injury; in the Netherlands, these figures were 56.8% and 27.0%, respectively (see Supporting Information: Sections S1.9–S1.12).

### 3.5 Sensitivity Analyses

ITS models with different permissible gap definitions, exclusion of data after the start of the COVID‐19 pandemic, and inclusion of seasonal trends were mostly consistent with the main analysis for fluoroquinolones but varied for other antibiotic groups. Exploration of different post‐intervention lag periods showed a greater immediate impact with shorter lags and greater change in trends with more longer lags (see Supporting Information: Section S1.13–S1.19).

## 4 Discussion

In this interrupted time series analysis study, we assessed the impact of RMMs targeting fluoroquinolone use, implemented in 2018/19 and 2020, on antibiotic use in primary care in the UK and the Netherlands. After the 2018/19 RMMs, a steady decrease was observed in both countries, with no further decrease after the second RMMs in 2020. The prescribing trends of alternative antibiotics following the 2018/19 RMMs were increasing for other antibacterials (J01XX) in both countries, but most drug classes had decreasing trends. Trends of most antibiotics were increasing post‐2020 RMMs.

Previous studies reported varying impacts of RMMs on fluoroquinolone use. An impact assessment in six EU countries, including the UK and the Netherlands, concluded that 2018/19 RMMs were not associated with significant changes in fluoroquinolone prescribing in primary care despite an overall reduction in fluoroquinolone prescribing in the UK [8]. A study in the Estonian outpatient population detected a declining trend of fluoroquinolone use prior to RMMs but suggested that RMMs might have contributed to a steeper decrease afterwards [9]. Similarly, a declining trend of prescribing fluoroquinolones in hospitals in the United States was reported following the 2016 black box warnings [20]. This is in line with our findings, which show a decreasing trend in prescribing fluoroquinolones following the 2018/19 RMMs. However, we observed opposite trends following the 2020 RMMs. Different incident use definitions could explain differences between our results and those of Ly et al.—we considered incident use only in patients not exposed to any antibiotics in the last 30 days, not only fluoroquinolones. Moreover, we used different data sources and different methods for ITS, such as generalized linear models versus joinpoint regression, and our study period was longer for CPRD data than in the previous studies.

We observed a downward shift in trends of some other antibiotic prescribing after the 2018/19 RMMs. Unlike in a German study, we did not detect a sustained increase in alternative antibiotic prescriptions after the dissemination of DHPCs [7]. While we saw a continuous rise in prescribing other antimicrobial drugs (under ATC code J01X) in both countries, in absolute numbers, the use of these drugs remained low and insufficient to substitute lower fluoroquinolone use. After 2020 RMMs, there was a gradual increase in prescribing some antibiotic groups; however, this rise did not coincide with decrease in fluoroquinolone use.

The association between 2018/19 RMMs and the decreasing prescribing of various antibiotic groups and opposite trends following the implementation of RMMs in 2020 suggests factors other than RMMs impacting prescribing patterns. The decreasing trends of other antibiotic use patterns might be attributed to increasing attention to antimicrobial resistance and international and national initiatives to improve antibiotic prescribing practices, such as the European One Health Action Plan launched in 2017 [21]. These policies could also have contributed to the changes in fluoroquinolone use patterns.

The upward shift in antibiotic prescribing after 2020 RMMs is unlikely to be related to RMMs. For fluoroquinolones, the increasing trends are mostly of lesser magnitude than decreasing trends after the 2018/19 RMMs, indicating a possible stabilization of fluoroquinolone prescription rates. However, the COVID‐19 pandemic may have been associated with interrupted healthcare provision and increased antibiotic prescribing [22, 23]. The pandemic might explain the sharp upward trend shift after 2020 RMMs, which is more prominent in PHARMO data where the post‐pandemic follow‐up was shorter [24]. A sensitivity analysis excluding data after the start of the COVID‐19 pandemic still suggests the impact of 2018/19 RMMs on fluoroquinolone prescribing but eliminates the possibility of assessing 2020 RMMs.

Factors beyond RMMs, such as heightened awareness of fluoroquinolone‐related adverse events and public health initiatives, may also explain these trends. Before implementing RMMs, EMA organized a public hearing on quinolone and fluoroquinolone antibiotics in June 2018 involving various stakeholders [25]. Also, the US FDA (Food and Drug Administration) issued a drug safety communication targeting fluoroquinolones in 2016, 2 years prior to RMMs issued by EMA [26].

Diverse impact estimates can be due to differing baseline prescribing rates before RMMs [27]. For example, no impact was observed in the previously mentioned multinational study in Belgium after 2018/19 RMMs, possibly due to the drop in prescribing after fluoroquinolone reimbursement changes in early 2018 [8, 28]. However, low baseline use does not always impede the ability to capture RMMs impact. Fluoroquinolone use assessed by indications in France, the UK, and Germany showed the strongest link to RMMs impact in the UK, which already had the lowest prescribing rates [10]. These national trends might further complicate the direct comparison of impact assessment results.

As broad‐spectrum antibiotics, fluoroquinolones are occasionally prescribed “just in case” to anticipate potential future ailments, such as traveler's diarrhea [29, 30]. This type of use was addressed during the EMA's referral procedure that prompted the 2018/19 RMMs [4]. Along with EU‐wide efforts in 2017 to combat antibiotic overuse, it may have influenced the prescribing patterns of fluoroquinolones and other antibiotics. However, local antibiotic resistance and availability variations might lead to prescribing practices that differ from international guidelines [31, 32]. Therefore, unlike in the case of market withdrawal, we expect that a group of patients will need fluoroquinolones after RMMs, though the optimal usage rate is unknown.

Our study had several strengths compared to other studies assessing the impact of RMMs targeting fluoroquinolones. First, beginning our follow‐up in 2014 allowed for more precise baseline antibiotic prescribing patterns before RMMs. Second, our study included data after the 2020 RMMs; the follow‐up period was longer for CPRD data than in previous studies. Third, since fluoroquinolones are seldom recommended as the first‐choice treatment, we assessed fluoroquinolone use in the treatment episodes of any antibiotic. This approach allowed us to check if reducing incident fluoroquinolone use can increase add‐on and continued use rates. Last, we combined RMMs and related events into two periods, allowing us to assess trends before and after complex interventions instead of focusing on a month‐to‐month basis.

While our study provides valuable insights, it is not without limitations. Challenges in identifying plausible indications for antibiotic prescriptions and potential confounding factors, such as the COVID‐19 pandemic, may have influenced our results. The decrease in fluoroquinolone use observed in our study might not fully represent the impact of regulatory measures, as it only examines drug use and does not capture potential adverse effects or health outcomes influenced by alternative treatments. Grouping multiple regulatory events in our definition of RMMs might conceal the impact of individual measures. However, individual assessment is complicated because there are not enough data points between them, and there are possible lag effects. Additionally, the impact of antibiotic use in secondary care remains unknown.

Further investigation is needed to assess the RMMs' effectiveness in promoting appropriate fluoroquinolone use in the broader context of clinical practice amidst rising antimicrobial resistance. RMMs are meant to improve health outcomes by minimizing the risk of adverse reactions related to drug use. While in our study, the decreasing use of fluoroquinolone (with no apparent increase in other antibiotics) might indicate the effectiveness of RMMs, observed drug use might not directly translate to health benefits, and the question remains whether RMMs are effective in improving health outcomes in individual patients. Future research should aim to measure the impact of RMMs on health outcomes.

## 5 Conclusion

The 2018/19 risk minimization measures (RMMs) targeting fluoroquinolones were accompanied by a moderate decrease in their use in primary care in the UK and the Netherlands. No further reduction was seen after 2020 RMMs. No sustained increase in alternative antibiotic prescribing was observed after the 2018/19, suggesting negligible unintended impact on other antibiotic overprescribing. However, the health benefits of avoiding harm associated with adverse events of fluoroquinolones remain unknown.

### 5.1 Plain Language Summary

Fluoroquinolones are a class of antibiotics used to treat various infections. Despite their effectiveness, the European Medicines Agency (EMA) further investigated the adverse effects of fluoroquinolones and found that they are associated with potentially severe and irreversible adverse effects affecting tendons and the cardiovascular system. These results led to new regulatory measures in 2018/2019 and again in 2020. These measures aim to increase awareness of the side effects among prescribers and improve the prescribing practices of antibiotics. We assessed the impact of these interventions on fluoroquinolone and other antibiotic prescribing patterns in the United Kingdom (UK) using the CPRD GOLD data and the Netherlands (NL) using PHARMO data. In our study, we included 4.0 million (UK) and 0.9 million (NL) antibiotic users from 2014 until 2021 (NL) or until 2023 (UK). We conducted interrupted time series analyses, a method that examines changes in trends over time before and after a specific intervention or event. Our results showed a decrease in fluoroquinolone prescribing immediately post‐2018/19 risk minimization measures (RMMs) and were followed by a steady decline. After the 2020 RMMS, no further reduction in fluoroquinolone prescribing was seen. Prescribing other antibiotics after the 2018/19 RMMs also mostly declined. Findings do not suggest unintended overprescribing of other antibiotics. However, the health benefits of these changes in antibiotic prescribing remain unknown.

## Ethics Statement

The authors have nothing to report.

## Conflicts of Interest

The authors declare no conflicts of interest.

## Supporting information


Data S1.

Data S2.

## Data Availability

The authors cannot share raw data from the CPRD GOLD or the PHARMO Data Network in the public domain. The data transformation and analysis scripts are publicly available on the OSF.io repository [33].

## References

[1] P. P. Majalekar and P. J. Shirote , “Fluoroquinolones: Blessings or Curses,” Current Drug Targets 21, no. 13 (2020): 1354–1370, 10.2174/1389450121666200621193355. 32564750

[2] H. H. Liu , “Safety Profile of the Fluoroquinolones,” Drug Safety 33, no. 5 (2010): 353–369, 10.2165/11536360-000000000-00000. 20397737

[3] R. Mehrzad and M. Barza , “Weighing the Adverse Cardiac Effects of Fluoroquinolones: A Risk Perspective,” Journal of Clinical Pharmacology 55, no. 11 (2015): 1198–1206, 10.1002/jcph.553. 26011799

[4] European Medicines Agency , “Assessment Report. Referral Under Article 31 of Directive 2001/83/EC Resulting From Pharmacovigilance Data: Quinolone and Fluoroquinolone Medicinal Products for Systemic and Inhalation Use (EMA/818158/2018),” 2018, https://www.ema.europa.eu/en/documents/referral/quinolone‐fluoroquinolone‐article‐31‐referral‐assessment‐report_en.pdf.

[5] European Medicines Agency , “Pharmacovigilance Risk Assessment Committee (PRAC) Recommendation. Fluoroquinolone and Quinolone Antibiotics: PRAC Recommends Restrictions on Use (EMA/668915/2018),” 2018, https://www.ema.europa.eu/en/documents/referral/quinolone‐fluoroquinolone‐article‐31‐referral‐prac‐recommends‐restrictions‐use_en.pdf.

[6] European Medicines Agency , “Direct Healthcare Professional Communication. Systemic and Inhaled Fluoroquinolones: Risk of Heart Valve Regurgitation/Incompetence,” 2020, https://www.ema.europa.eu/en/documents/dhpc/direct‐healthcare‐professional‐communication‐dhpc‐systemic‐inhaled‐fluoroquinolones‐risk‐heart‐valve/incompetence_en.pdf.

[7] U. Georgi , F. Tesch , J. Schmitt , and K. de With , “Impact of Safety Warnings for Fluoroquinolones on Prescribing Behaviour. Results of a Cohort Study With Outpatient Routine Data,” Infection 49, no. 3 (2021): 447–455, 10.1007/s15010-020-01549-7. 33258075 PMC8159769

[8] N. F. Ly , C. Flach , T. S. Lysen , et al., “Impact of European Union Label Changes for Fluoroquinolone‐Containing Medicinal Products for Systemic and Inhalation Use: Post‐Referral Prescribing Trends,” Drug Safety 46 (March 2023): 405–416, 10.1007/s40264-023-01286-4. 36976448 PMC10044099

[9] K. Kurvits , M. Uusküla , H. Vestman , and O. Laius , “Outpatient Fluoroquinolone Use in Relation to European Medicines Agency's Recommendation: An Estonian Nationwide Drug Utilization Study,” Pharmacoepidemiology and Drug 32, no. 6 (February 2023): 643–650, 10.1002/pds.5593. 36690579

[10] D. R. Morales , J. Slattery , L. Pinheiro , X. Kurz , and K. Hedenmalm , “Indications for Systemic Fluoroquinolone Therapy in Europe and Prevalence of Primary‐Care Prescribing in France, Germany and the UK: Descriptive Population‐Based Study,” Clinical Drug Investigation 38, no. 10 (2018): 927–933, 10.1007/s40261-018-0684-7. 30143952

[11] A. Machowska and L. C. Stålsby , “Drivers of Irrational Use of Antibiotics in Europe,” International Journal of Environmental Research and Public Health 16, no. 1 (2019): 27, 10.3390/ijerph16010027. PMC6338985 30583571

[12] E. Herrett , A. M. Gallagher , K. Bhaskaran , et al., “Data Resource Profile: Clinical Practice Research Datalink (CPRD),” International Journal of Epidemiology 44, no. 3 (2015): 827–836, 10.1093/ije/dyv098. 26050254 PMC4521131

[13] A. M. Gallagher , A. A. Kousoulis , T. Williams , J. Valentine , and P. Myles , “Clinical Practice Research Datalink (CPRD),” in Databases for Pharmacoepidemiological Research. Springer Series on Epidemiology and Public Health, eds. M. Sturkenboom and T. Schink (Cham, Switzerland: Springer International Publishing, 2021), 57–65, 10.1007/978-3-030-51455-6_3.

[14] Medicines & Healthcare products Regulatory Agency , “Clinical Practice Research Datalink (CPRD),” accessed March 25, 2024, www.cprd.com, http://www.cprd.com/.

[15] The PHARMO Institute , “The PHARMO Institute Website. pharmo.nl,” accessed October 12, 2023, https://pharmo.nl/pharmo‐data/.

[16] J. G. Kuiper , M. Bakker , F. J. A. Penning‐van Beest , and R. M. C. Herings , “Existing Data Sources for Clinical Epidemiology: The PHARMO Database Network,” Clinical Epidemiology 12 (2020): 415–422, 10.2147/CLEP.S247575. 32425609 PMC7196787

[17] WHO Collaborating Centre for Drug Statistics Methodology , “Guidelines for ATC Classification and DDD Assignment 2023,” 2022, https://atcddd.fhi.no/filearchive/publications/1_2023_guidelines__final_web.pdf.

[18] R. Jandoc , A. M. Burden , M. Mamdani , L. E. Lévesque , and S. M. Cadarette , “Interrupted Time Series Analysis in Drug Utilization Research Is Increasing: Systematic Review and Recommendations,” Journal of Clinical Epidemiology 68, no. 8 (2015): 950–956, 10.1016/j.jclinepi.2014.12.018. 25890805

[19] C. Cunningham , L. Fisher , C. Wood , et al., “Incidence and Treatment of Group A Streptococcal Infections During Covid‐19 Pandemic and 2022 Outbreak: Retrospective Cohort Study in England Using OpenSAFELY‐TPP,” British Medical Journal Medicine 3, no. 1 (2024): e000791, 10.1136/bmjmed-2023-000791. 38803829 PMC11129040

[20] M. E. Yarrington , D. J. Anderson , E. D. Ashley , et al., “Impact of FDA Black Box Warning on Fluoroquinolone and Alternative Antibiotic Use in Southeastern US Hospitals,” Infection Control & Hospital Epidemiology 40, no. 11 (2019): 1297–1300, 10.1017/ice.2019.247. 31474240

[21] European Commission , “A European One Health Action Plan Against Antimicrobial Resistance (AMR),” 2017, https://health.ec.europa.eu/antimicrobial‐resistance/eu‐action‐antimicrobial‐resistance_en.

[22] T. M. Rawson , L. S. P. Moore , E. Castro‐Sanchez , et al., “COVID‐19 and the Potential Long‐Term Impact on Antimicrobial Resistance,” Journal of Antimicrobial Chemotherapy 75, no. 7 (2020): 1681–1684, 10.1093/jac/dkaa194. 32433765 PMC7314000

[23] G. Pujolar , A. Oliver‐Anglès , I. Vargas , and M. L. Vázquez , “Changes in Access to Health Services During the COVID‐19 Pandemic: A Scoping Review,” International Journal of Environmental Research and Public Health 19, no. 3 (2022): 1749, 10.3390/ijerph19031749. 35162772 PMC8834942

[24] R. Armitage and L. B. Nellums , “Antibiotic Prescribing in General Practice During COVID‐19,” Lancet Infectious Diseases 21, no. 6 (2021): e144, 10.1016/S1473-3099(20)30917-8. 33275941 PMC9761101

[25] European Medicines Agency , “Summary of the EMA Public Hearing on Quinolone and Fluoroquinolone Antibiotics. Public Hearing Held on 13 June 2018,” 2018, https://www.ema.europa.eu/en/documents/report/summary‐ema‐public‐hearing‐quinolone‐and‐fluoroquinolone‐antibiotics_en.pdf.

[26] U.S. Food and Drug Administration , “FDA Drug Safety Communication: FDA Advises Restricting Fluoroquinolone Antibiotic Use for Certain Uncomplicated Infections; Warns About Disabling Side Effects That Can Occur Together,” 2016, https://www.fda.gov/drugs/drug‐safety‐and‐availability/fda‐drug‐safety‐communication‐fda‐advises‐restricting‐fluoroquinolone‐antibiotic‐use‐certain.

[27] R. Brauer , A. Ruigómez , G. Downey , et al., “Prevalence of Antibiotic Use: A Comparison Across Various European Health Care Data Sources,” Pharmacoepidemiology and Drug Safety 25, no. Suppl 1 (2016): 11–20, 10.1002/pds.3831. PMC4918309 26152658

[28] H. Vermeulen , S. Coenen , N. Hens , and R. Bruyndonckx , “Impact of Changing Reimbursement Criteria on the Use of Fluoroquinolones in Belgium,” Journal of Antimicrobial Chemotherapy 76, no. 10 (2021): 2725–2732, 10.1093/jac/dkab255. 34374778 PMC8446932

[29] D. N. Taylor , D. H. Hamer , and D. R. Shlim , “Medications for the Prevention and Treatment of Travellers' Diarrhea,” Journal of Travel Medicine 24, no. suppl_1 (2017): S17–S22, 10.1093/jtm/taw097. 28520998

[30] C. J. McGow , “Prescribing Antibiotics “Just in Case” Must Be Tackled to Slow Rise in Antibiotic Resistance,” BMJ 364 (2019): l553, 10.1136/bmj.l553. 30728134

[31] S. Mayor , “Antibiotic Resistant Bacteria Cause Nearly One in Five Infections in Wealthy Countries, Report Warns,” BMJ: British Medical Journal 363 (2018): 363, https://www.jstor.org/stable/26963990. 10.1136/bmj.k4762 30409845

[32] N. Miljković , P. Polidori , and S. Kohl , “Managing Antibiotic Shortages: Lessons From EAHP and ECDC Surveys,” European Journal of Hospital Pharmacy 29, no. 2 (2022): 90–94, 10.1136/ejhpharm-2021-003110. 35190453 PMC8899686

[33] T. Lasys , Y. Santa‐Ana‐Tellez , S. J. Siiskonen , R. H. H. Groenwold , and H. Gardarsdottir , “Project Repository: Impact of Pharmacovigilance Interventions Targeting Fluoroquinolones On Antibiotic Use in the Netherlands and the United Kingdom,” 2024, 10.17605/OSF.IO/9JMCB. PMC11739677 39821460

