# Supplementary material for: Impact of Pharmacovigilance Interventions Targeting Fluoroquinolones on Antibiotic Use in the Netherlands and the United Kingdom
Source: Pharmacoepidemiol Drug Saf. 2025 Jan 16;34(2):e70081. doi: 10.1002/pds.70081 (PMC11739677; doi:10.1002/pds.70081)
Supplement: Supplementary file 1 — Data S1. [file PDS-34-e70081-s002.docx]

Impact of pharmacovigilance interventions targeting fluoroquinolones on antibiotic use in the Netherlands and the United Kingdom.

Authors: Tomas Lasys, Yared Santa-Ana-Tellez, Satu J. Siiskonen, Rolf H.H. Groenwold, Helga Gardarsdottir

Corresponding author: Helga Gardarsdottir, [h.gardarsdottir@uu.nl](mailto:h.gardarsdottir@uu.nl)

Supplementary material

[Additional information on study design 2](#_Toc183109683)

[S1.1. Summary of regulatory events concerning (fluoro)quinolone antibiotics during the study period 2](#_Toc183109684)

[S1.2. A graphical depiction of the study design and inclusion criteria 2](#_Toc183109685)

[S1.3. Graphical depiction of treatment episode construction and different categories of prescriptions (incident use, add-on use and continued use) 3](#_Toc183109686)

[S1.4. Models used in the analysis 4](#_Toc183109687)

[Additional descriptive statistics 5](#_Toc183109688)

[S1.5. Cohort details at index date 5](#_Toc183109689)

[S1.6. Risk factors at the start of each treatment episode 6](#_Toc183109690)

[Analysis of fluoroquinolone prescription patterns 7](#_Toc183109691)

[S1.7. Interrupted time series regression analysis for fluoroquinolone use patterns 7](#_Toc183109692)

[S1.8. Interrupted time series regression analysis of incident use prescription rates of antibiotics (ATC code J01) 9](#_Toc183109693)

[Covariate analyses for fluoroquinolone prescription patterns 11](#_Toc183109694)

[S1.9. Interrupted time series regression results for fluoroquinolone prescription rates for incident use stratified by sex 11](#_Toc183109695)

[S1.10. Interrupted time series regression results for fluoroquinolone prescription rates for incident use stratified by age group 12](#_Toc183109696)

[S1.11. Interrupted time series regression results for fluoroquinolone prescription rates for incident use stratified by indication 14](#_Toc183109697)

[S1.12. Interrupted time series regression results for fluoroquinolone prescription rates for incident use stratified by risk group 15](#_Toc183109698)

[Sensitivity analyses 16](#_Toc183109699)

[S1.13. Prescription rates of any antiobiotics (ATC J01) and fluoroquinolones for incident use depending on definition of permissible gap in constructing treatment episodes. 16](#_Toc183109700)

[S1.14. Interrupted time series regression analysis for fluoroquinolones depending on permissible gap length used in construction of treatment episodes and prescription type. 18](#_Toc183109701)

[S1.15. Interrupted time series regression analysis for incident use of fluoroquinolones considering different post-intervention lag periods. 20](#_Toc183109702)

[S1.16. Prescription rates incident use of fluoroquinolones and 5 of the most frequently used antibiotics (ATC code J01) for incident use: analysis excluding data after the start of the COVID-19 pandemic and without considering post-intervention lag period. 21](#_Toc183109703)

[S1.17. Interrupted time series regression analysis for antibiotics (ATC code J01) incident use rates excluding the data after the start of the COVID-19 pandemic from the model estimation (and no lag window). 23](#_Toc183109704)

[S1.18. Interrupted time series regression analysis for antibiotics (ATC code J01) incident use rates without exclusion of lag period from the model estimation 25](#_Toc183109705)

[S1.19. Interrupted time series regression analysis for antibiotics (ATC coode J01) incident use rates with inclusion of seasonal trends 27](#_Toc183109706)

# Additional information on study design

## Summary of regulatory events concerning (fluoro)quinolone antibiotics during the study period

|  | **Date** | **Regulatory event** |
| --- | --- | --- |
| 2018/19 RMMs  (for both the Netherlands and the UK) | 04 October 2018 | 'EMA's Pharmacovigilance Risk Assessment Committee (PRAC) recommends restrictions on the use of fluoroquinolone and quinolone antibiotics |
|  | 15 November 2018 | 'EMA's Committee for Medicinal Products for Human Use (CHMP) accepts 'PRAC's recommendations and forwards their opinion to the European Commission (EC) |
|  | 14 February 2019 | EC issues a binding decision to implement RMMs targeting Quinsar (inhaled levofloxacin) |
|  | 11 March 2019 | EC issues a binding decision to implement RMMs targeting all other quinolone and fluoroquinolone antibiotics |
|  | 21 March 2019 | DHPC issued in the UK |
|  | 29 March 2019 | DHPC issued in the Netherlands |
| 2020 RMMs  (for the Netherlands) | 28 September 2020 | PRAC recommends to amend the  product information for fluoroquinolones for systemic and inhalation formulations by including information about the risk of heart valve  regurgitation and heart valve incompetence |
|  | 29 October 2020 | DHPC issued by EMA |
|  | 29 October 2020 | DHPC issued in the Netherlands |
| 2020 RMMs’  (for the UK) | 2 December 2020 | DHPC issued in the UK |

EMA European Medicines Agency, PRAC Pharmacovigilance Risk Assessment Committee, EC European Commission, DHPC Direct Healthcare Professional Communication, UK United Kingdom, RMMs Risk minimization measures.

## A graphical depiction of the study design and inclusion criteria


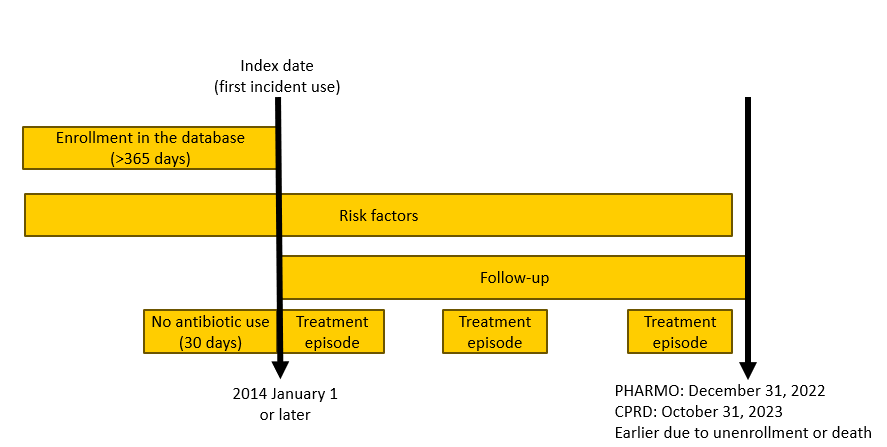


Subjects were enrolled in the cohort on the study start date (January 1 2014), or at the time of the first antibiotic prescription after they were enrolled in the database for at least 365 days and had at least 365 days antibiotic free window. Subjects were followed up until the study end (31 December 2021 for PHARMO and 31 October 2023 for CPRD), or loss to follow-up due to any reason. After the first entry, multiple treatment episodes per subject were permitted. Treatments occurring within up to 30 days between treatment days (permissible gap) were considered as part of the same treatment episode. If the gap was longer than 30 days, a new prescription would be considered to belong to a new treatment episode. Permissible gaps of 7, 10, and 14 days were explored in sensitivity analysis.

## Graphical depiction of treatment episode construction and different categories of prescriptions (incident use, add-on use and continued use)


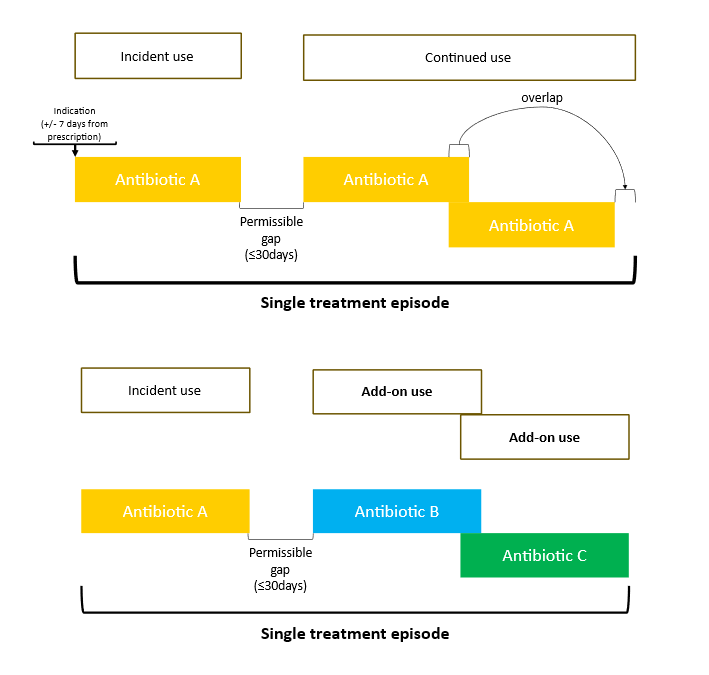


Treatment episodes were constructed by estimating the duration of each prescription, calculated from the start date to the estimated end date. This end date was determined by dividing the quantity of medicine prescribed by the treatment regimen. The standard defined daily dose (DDD) was used if the information on the treatment regimen was unavailable. Any subsequent antibiotic prescriptions within a 30-day permissible gap after the estimated end date of the prior prescription were considered part of the same treatment episode (continued use). Overlapping periods of prescriptions of the same product were appended to the end of the last prescription.

Prescriptions were categorized as incident, add-on, or continued use. The first prescription in the treatment episode was classified as incident use. Subsequent prescriptions were considered treatment continuation if the same antibiotic was prescribed or treatment add-on if a different antibiotic was prescribed during the same treatment episode. The term add-on was used because, due to the typically short treatment durations for antibiotics (usually requiring one prescription) and the lack of diagnosis information, verifying whether another antibiotic was used as a switch for the same indication might not be possible.

## Models used in the analysis


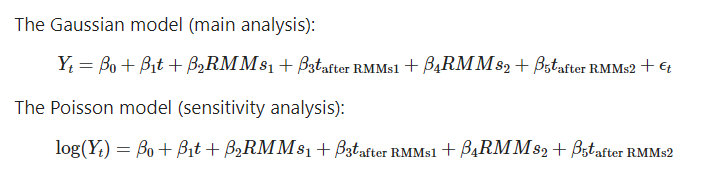


In both models, the main outcome (*Y_t_*) is the rate of monthly prescriptions per 10,000 person-years at the time *t* (in months) calculated by multiplying the number of prescriptions in that month by 10,000 and dividing by the total number of person-years contributed to the database that month.

*β_0_­* - the intercept term. It represents the baseline prescription rate when all other variables are zero.

*β_1_t­* - the pre-intervention slope (*t* is the time in months after the start of the study), and captures the underlying trend in the prescription rate over time before any interventions.

*β_1_­RMMs_1_* - the immediate change in the prescription rate following the 2018/19 Risk Minimization Measures (RMMs). *RMMs_1_* is a binary indicator variable (0 before the intervention, 1 after the intervention).

*β_3_t_after RMMs1_­* - The change in the slope of the prescription rate after the 2018/19 RMMs.

*β_3_­RMMs_2_* - the immediate change in the prescription rate following the 2020 Risk Minimization Measures (RMMs). *RMMs_2_* is a binary indicator variable (0 before the intervention, 1 after the intervention).

*β_4_t_after RMMs2_* - the change in the slope of the prescription rate after the 2020 RMMs.

*ε_t_* - The error term. It captures the random variability in the prescription rate that is not explained by the model.

# Additional descriptive statistics

## Cohort details at index date

| variable | description | CPRD | PHARMO |
| --- | --- | --- | --- |
| Age group | <2 years | 83,325 ( 2.1%) | 5,431 ( 0.6%) |
|  | 2 to <12 years | 533,872 (13.3%) | 85,561 ( 9.8%) |
|  | 12 to <19 years | 271,859 ( 6.8%) | 56,425 ( 6.5%) |
|  | 19 to <30 years | 476,982 (11.9%) | 100,750 (11.6%) |
|  | 30 to <40 years | 494,855 (12.4%) | 93,951 (10.8%) |
|  | 40 to <50 years | 494,643 (12.4%) | 115,033 (13.2%) |
|  | 50 to <60 years | 518,914 (13.0%) | 135,369 (15.6%) |
|  | 60 to <70 years | 468,763 (11.7%) | 130,098 (15.0%) |
|  | 70 to <80 years | 371,110 ( 9.3%) | 92,904 (10.7%) |
|  | 80 years and older | 285,218 ( 7.1%) | 54,055 ( 6.2%) |
| Lookback period from registration to the database until inclusion (in months) | Q1 | 35.0 | 48.4 |
|  | Median | 108.0 | 81.7 |
|  | Mean | 109.4 | 84.8 |
|  | Q3 | 164.9 | 118.8 |
| Risk factors^a^ | no known risk factors | 2,206,169 (55.2%) | 581,463 (66.9%) |
|  | aortic aneurysm | 9,193 ( 0.2%) | 9,918 ( 1.1%) |
|  | aortic valve disorder | 12,154 ( 0.3%) | 9,037 ( 1.0%) |
|  | cerebrovascular diseases | 59,471 ( 1.5%) | 21,267 ( 2.4%) |
|  | concomitant glucocorticoid use | 177,606 ( 4.4%) | 34,117 ( 3.9%) |
|  | dislipidemia | 152,135 ( 3.8%) | 72,997 ( 8.4%) |
|  | dissection of aorta | 1,192 (<0.1%) | 189 (<0.1%) |
|  | hypertension | 586,274 (14.7%) | 112,841 (13.0%) |
|  | ischemic heart diseases | 159,003 ( 4.0%) | 37,017 ( 4.3%) |
|  | prior lipid-lowering medication use | 742,472 (18.6%) | 148,147 (17.0%) |
|  | renal impairment | 185,307 ( 4.6%) | 9,328 ( 1.1%) |
|  | solid organ transplant | 2,917 ( 0.1%) | 5,259 ( 0.6%) |
|  | tendinitis | 149,208 ( 3.7%) | 17,223 ( 2.0%) |
|  | tendon rupture | 4,154 ( 0.1%) | 204 (<0.1%) |
|  | prior tobacco use | 955,851 (23.9%) | 33,977 ( 3.9%) |
| Cohort entry year | 2014 | 1,528,386 (38.2%) | 229,175 (26.4%) |
|  | 2015 | 702,455 (17.6%) | 162,200 (18.7%) |
|  | 2016 | 433,119 (10.8%) | 125,772 (14.5%) |
|  | 2017 | 308,362 ( 7.7%) | 101,546 (11.7%) |
|  | 2018 | 251,582 ( 6.3%) | 89,391 (10.3%) |
|  | 2019 | 215,534 ( 5.4%) | 73,753 ( 8.5%) |
|  | 2020 | 142,234 ( 3.6%) | 49,298 ( 5.7%) |
|  | 2021 | 139,719 ( 3.5%) | 38,442 ( 4.4%) |
|  | 2022 | 159,718 ( 4.0%) | NA |
|  | 2023 | 118,432 ( 3.0%) | NA |

^a^in the interrupted time series analysis, the presence of risk was assessed at the start of each treatment episode.

Risk factors were grouped based on risk factors specified in Direct Healthcare Professional Communications: 1) Risk factors for aortic aneurysm or dissection: history of aortic aneurysm, aortic valve disorder, cerebrovascular disease, dyslipidaemia, dissection of aorta, hypertension, ischaemic heart disease, prior lipid-lowering medication use or prior tobacco use; 2) Risk factors for tendonitis or tendon rupture: concomitant glucocorticoid use, renal impairment, solid organ transplant, tendinitis, or prior tobacco use.

## Risk factors at the start of each treatment episode

| **variable** | **description** | **CPRD** | **PHARMO** |
| --- | --- | --- | --- |
| Risk factors | no known risk factors | 5,556,972 (40.7%) | 1,212,148 (54.3%) |
|  | aortic aneurysm | 65,520 ( 0.5%) | 44,604 ( 2.0%) |
|  | aortic valve disorder | 73,144 ( 0.5%) | 44,501 ( 2.0%) |
|  | cerebrovascular diseases | 358,717 ( 2.6%) | 115,810 ( 5.2%) |
|  | concomitant glucocorticoid use | 1,249,036 ( 9.1%) | 149,783 ( 6.7%) |
|  | dislipidemia | 871,585 ( 6.4%) | 195,367 ( 8.8%) |
|  | dissection of aorta | 1,192 (<0.1%) | 1,032 (<0.1%) |
|  | hypertension | 3,195,706 (23.4%) | 426,791 (19.1%) |
|  | ischemic heart diseases | 911,747 ( 6.7%) | 156,692 ( 7.0%) |
|  | prior lipid-lowering medication use | 3,702,930 (27.1%) | 540,205 (24.2%) |
|  | renal impairment | 1,256,103 ( 9.2%) | 121,752 ( 5.5%) |
|  | solid organ transplant | 29,889 ( 0.2%) | 26,809 ( 1.2%) |
|  | tendinitis | 780,997 ( 5.7%) | 108,984 ( 4.9%) |
|  | tendon rupture | 21,604 ( 0.2%) | 666 (<0.1%) |
|  | prior tobacco use | 10,169,326 (74.4%) | 256,538 (11.5%) |
| Risk group^a^ | Risk factors for aortic aneurysm or dissection | 7,600,108 (55.6%) | 895,821 (40.1%) |
|  | Risk factors for tendonitis or tendon rupture | 6,510,138 (47.6%) | 460,027 (20.6%) |
|  | No known risk factors | 5,556,972 (40.7%) | 1,212,148 (54.3%) |

^a^in the interrupted time series analysis the presence of risk was assessed at the start of each treatment episode.

Risk factors were grouped based on risk factors specified in Direct Healthcare Professional Communications: 1) Risk factors for aortic aneurysm or dissection: history of aortic aneurysm, aortic valve disorder, cerebrovascular disease, dyslipidaemia, dissection of aorta, hypertension, ischaemic heart disease, prior lipid-lowering medication use or prior tobacco use; 2) Risk factors for tendonitis or tendon rupture: concomitant glucocorticoid use, renal impairment, solid organ transplant, tendinitis, or prior tobacco use.

# Analysis of fluoroquinolone prescription patterns

## Interrupted time series regression analysis for fluoroquinolone use patterns

| Database | Drug | Prescription type | (Intercept)  [95% CI] | Slope before RMMs  [95% CI] | Step change after 2018/19 RMMs [95% CI] | Slope change after 2018/19 RMMs  [95% CI] | Step change after 2020 RMMs  [95% CI] | Slope change after 2020 RMMs  [95% CI] |
| --- | --- | --- | --- | --- | --- | --- | --- | --- |
| CPRD-UK | ciprofloxacin | incident | **57.692 [56.312 to 59.072]  p < 0.001** | **-0.125 [-0.165 to -0.086]  p < 0.001** | **-4.858 [-8.258 to -1.459]  p = 0.006** | **-0.614 [-0.845 to -0.382]  p < 0.001** | 2.684 [-0.491 to 5.859]  p = 0.101 | **0.565 [ 0.314 to 0.815]  p < 0.001** |
| CPRD-UK | ciprofloxacin | add-on | **35.101 [33.545 to 36.657]  p < 0.001** | 0.025 [-0.019 to 0.069]  p = 0.268 | **-6.140 [-9.973 to -2.307]  p = 0.002** | **-0.395 [-0.656 to -0.134]  p = 0.004** | 0.359 [-3.221 to 3.938]  p = 0.845 | 0.277 [-0.004 to 0.559]  p = 0.056 |
| CPRD-UK | ciprofloxacin | continued | **17.355 [16.396 to 18.314]  p < 0.001** | **0.071 [ 0.043 to 0.098]  p < 0.001** | **-4.647 [-7.009 to -2.285]  p < 0.001** | **-0.170 [-0.330 to -0.009]  p = 0.041** | -0.223 [-2.429 to 1.982]  p = 0.843 | -0.029 [-0.203 to 0.145]  p = 0.744 |
| CPRD-UK | levofloxacin | incident | **0.770 [ 0.601 to 0.939]  p < 0.001** | **0.019 [ 0.014 to 0.024]  p < 0.001** | 0.008 [-0.408 to 0.424]  p = 0.972 | -0.020 [-0.048 to 0.008]  p = 0.169 | 0.342 [-0.046 to 0.731]  p = 0.087 | 0.021 [-0.010 to 0.052]  p = 0.180 |
| CPRD-UK | levofloxacin | add-on | **0.890 [ 0.729 to 1.050]  p < 0.001** | **0.019 [ 0.015 to 0.024]  p < 0.001** | -0.123 [-0.518 to 0.272]  p = 0.544 | **-0.060 [-0.087 to -0.033]  p < 0.001** | 0.108 [-0.261 to 0.477]  p = 0.567 | **0.054 [ 0.025 to 0.083]  p < 0.001** |
| CPRD-UK | levofloxacin | continued | **0.350 [ 0.263 to 0.438]  p < 0.001** | **0.015 [ 0.012 to 0.017]  p < 0.001** | 0.010 [-0.205 to 0.225]  p = 0.929 | **-0.018 [-0.033 to -0.004]  p = 0.015** | -0.183 [-0.383 to 0.018]  p = 0.078 | -0.004 [-0.020 to 0.011]  p = 0.583 |
| CPRD-UK | ofloxacin | incident | **4.650 [ 4.304 to 4.996]  p < 0.001** | **0.039 [ 0.029 to 0.049]  p < 0.001** | **-1.066 [-1.918 to -0.213]  p = 0.016** | -0.051 [-0.109 to 0.007]  p = 0.089 | 0.722 [-0.074 to 1.518]  p = 0.079 | -0.021 [-0.084 to 0.042]  p = 0.511 |
| CPRD-UK | ofloxacin | add-on | **1.217 [ 1.095 to 1.339]  p < 0.001** | **0.008 [ 0.005 to 0.012]  p < 0.001** | **-0.437 [-0.738 to -0.136]  p = 0.005** | 0.001 [-0.020 to 0.021]  p = 0.934 | 0.010 [-0.271 to 0.291]  p = 0.947 | -0.009 [-0.031 to 0.013]  p = 0.429 |
| CPRD-UK | ofloxacin | continued | **0.803 [ 0.691 to 0.914]  p < 0.001** | **0.003 [ 0.000 to 0.006]  p = 0.045** | -0.083 [-0.358 to 0.191]  p = 0.553 | -0.003 [-0.022 to 0.015]  p = 0.732 | -0.017 [-0.273 to 0.240]  p = 0.899 | -0.004 [-0.024 to 0.016]  p = 0.686 |
| PHARMO-NL | ciprofloxacin | incident | **62.719 [ 58.527 to 66.912]  p < 0.001** | **0.700 [ 0.586 to 0.813]  p < 0.001** | **-18.324 [-27.179 to -9.469]  p < 0.001** | **-0.847 [ -1.545 to -0.149]  p = 0.020** | -7.839 [-19.619 to 3.942]  p = 0.196 | 1.180 [ -0.109 to 2.469]  p = 0.076 |
| PHARMO-NL | ciprofloxacin | add-on | **18.746 [ 16.642 to 20.849]  p < 0.001** | **0.388 [ 0.331 to 0.445]  p < 0.001** | **-7.906 [-12.348 to -3.464]  p = 0.001** | -0.320 [ -0.670 to 0.031]  p = 0.077 | -4.660 [-10.570 to 1.250]  p = 0.126 | 0.536 [ -0.111 to 1.183]  p = 0.108 |
| PHARMO-NL | ciprofloxacin | continued | **9.026 [ 8.084 to 9.967]  p < 0.001** | **0.130 [ 0.104 to 0.155]  p < 0.001** | **-4.570 [-6.558 to -2.581]  p < 0.001** | -0.032 [-0.189 to 0.124]  p = 0.688 | -1.455 [-4.100 to 1.190]  p = 0.284 | 0.072 [-0.218 to 0.361]  p = 0.629 |
| PHARMO-NL | levofloxacin | incident | **1.998 [ 1.546 to 2.451]  p < 0.001** | **0.087 [ 0.075 to 0.100]  p < 0.001** | 0.855 [-0.100 to 1.809]  p = 0.083 | **-0.162 [-0.237 to -0.086]  p < 0.001** | 0.765 [-0.505 to 2.036]  p = 0.241 | **0.166 [ 0.027 to 0.305]  p = 0.021** |
| PHARMO-NL | levofloxacin | add-on | **0.467 [ 0.315 to 0.619]  p < 0.001** | **0.009 [ 0.005 to 0.013]  p < 0.001** | 0.037 [-0.284 to 0.358]  p = 0.821 | -0.019 [-0.044 to 0.006]  p = 0.146 | -0.122 [-0.549 to 0.305]  p = 0.577 | 0.036 [-0.011 to 0.083]  p = 0.135 |
| PHARMO-NL | levofloxacin | continued | **0.308 [ 0.163 to 0.454]  p < 0.001** | **0.007 [ 0.003 to 0.011]  p = 0.001** | -0.055 [-0.363 to 0.252]  p = 0.724 | 0.016 [-0.008 to 0.041]  p = 0.191 | 0.132 [-0.277 to 0.540]  p = 0.530 | **-0.054 [-0.099 to -0.009]  p = 0.020** |
| PHARMO-NL | moxifloxacin | incident | **1.997 [ 1.703 to 2.291]  p < 0.001** | -0.003 [-0.011 to 0.005]  p = 0.439 | -0.326 [-0.948 to 0.295]  p = 0.306 | -0.010 [-0.059 to 0.039]  p = 0.704 | -0.391 [-1.218 to 0.435]  p = 0.356 | 0.088 [-0.002 to 0.179]  p = 0.059 |
| PHARMO-NL | moxifloxacin | add-on | **1.030 [ 0.786 to 1.274]  p < 0.001** | **0.007 [ 0.000 to 0.014]  p = 0.045** | -0.210 [-0.726 to 0.305]  p = 0.426 | -0.031 [-0.072 to 0.009]  p = 0.134 | 0.012 [-0.674 to 0.698]  p = 0.973 | 0.022 [-0.053 to 0.097]  p = 0.565 |
| PHARMO-NL | moxifloxacin | continued | **0.391 [ 0.279 to 0.503]  p < 0.001** | 0.001 [-0.002 to 0.004]  p = 0.521 | -0.109 [-0.346 to 0.128]  p = 0.370 | -0.006 [-0.024 to 0.013]  p = 0.560 | -0.067 [-0.382 to 0.248]  p = 0.678 | **0.044 [ 0.010 to 0.079]  p = 0.013** |
| PHARMO-NL | norfloxacin | incident | **5.921 [ 5.509 to 6.333]  p < 0.001** | **-0.048 [-0.060 to -0.037]  p < 0.001** | -0.700 [-1.570 to 0.171]  p = 0.119 | 0.037 [-0.032 to 0.105]  p = 0.296 | 0.165 [-0.993 to 1.323]  p = 0.780 | -0.030 [-0.157 to 0.097]  p = 0.645 |
| PHARMO-NL | norfloxacin | add-on | **2.943 [ 2.667 to 3.219]  p < 0.001** | **-0.027 [-0.034 to -0.020]  p < 0.001** | -0.203 [-0.785 to 0.380]  p = 0.497 | 0.018 [-0.028 to 0.064]  p = 0.442 | -0.082 [-0.857 to 0.692]  p = 0.836 | 0.008 [-0.076 to 0.093]  p = 0.847 |
| PHARMO-NL | norfloxacin | continued | **0.723 [ 0.540 to 0.906]  p < 0.001** | **0.016 [ 0.011 to 0.021]  p < 0.001** | **-0.599 [-0.986 to -0.213]  p = 0.003** | 0.000 [-0.031 to 0.030]  p = 0.985 | -0.026 [-0.540 to 0.488]  p = 0.921 | 0.011 [-0.045 to 0.067]  p = 0.705 |
| PHARMO-NL | ofloxacin | incident | **2.527 [ 2.301 to 2.753]  p < 0.001** | 0.004 [-0.003 to 0.010]  p = 0.251 | 0.165 [-0.312 to 0.641]  p = 0.499 | **-0.064 [-0.102 to -0.027]  p = 0.001** | **0.682 [ 0.048 to 1.316]  p = 0.038** | -0.006 [-0.075 to 0.064]  p = 0.868 |
| PHARMO-NL | ofloxacin | add-on | **0.479 [ 0.354 to 0.604]  p < 0.001** | **0.004 [ 0.001 to 0.008]  p = 0.012** | **-0.278 [-0.542 to -0.013]  p = 0.042** | -0.003 [-0.024 to 0.017]  p = 0.751 | 0.210 [-0.142 to 0.562]  p = 0.244 | -0.031 [-0.070 to 0.007]  p = 0.118 |
| PHARMO-NL | ofloxacin | continued | **0.232 [ 0.157 to 0.308]  p < 0.001** | 0.000 [-0.002 to 0.002]  p = 0.765 | 0.001 [-0.158 to 0.161]  p = 0.986 | -0.007 [-0.020 to 0.005]  p = 0.258 | 0.071 [-0.142 to 0.283]  p = 0.516 | 0.007 [-0.016 to 0.030]  p = 0.552 |

Products with low cell counts (> 10% cells of monthly counts with values of 5 or less) were excluded.

## Interrupted time series regression analysis of incident use prescription rates of antibiotics (ATC code J01)

| Database | Drug class | (Intercept)  [95% CI] | Slope before RMMs  [95% CI] | Step change after 2018/19 RMMs [95% CI] | Slope change after 2018/19 RMMs  [95% CI] | Step change after 2020 RMMs  [95% CI] | Slope change after 2020 RMMs  [95% CI] |
| --- | --- | --- | --- | --- | --- | --- | --- |
| CPRD-UK | Beta-lactamase resistant penicillins | **503.194 [477.191 to 529.196]  p < 0.001** | -0.321 [ -1.061 to 0.418]  p = 0.396 | 9.096 [-54.969 to 73.162]  p = 0.781 | -0.679 [ -5.041 to 3.682]  p = 0.761 | 2.453 [-57.381 to 62.286]  p = 0.936 | 1.355 [ -3.358 to 6.067]  p = 0.574 |
| CPRD-UK | Beta-lactamase sensitive penicillins | **271.386 [227.630 to 315.142]  p < 0.001** | -0.284 [ -1.529 to 0.960]  p = 0.656 | 46.163 [-61.643 to 153.970]  p = 0.403 | -6.831 [-14.170 to 0.509]  p = 0.071 | 53.470 [-47.215 to 154.155]  p = 0.300 | **12.483 [ 4.553 to 20.413]  p = 0.003** |
| CPRD-UK | Combinations of penicillins | **154.381 [150.224 to 158.538]  p < 0.001** | **-0.927 [ -1.045 to -0.809]  p < 0.001** | 4.993 [ -5.250 to 15.235]  p = 0.342 | 0.638 [ -0.059 to 1.335]  p = 0.076 | 5.716 [ -3.850 to 15.282]  p = 0.244 | 0.493 [ -0.260 to 1.246]  p = 0.203 |
| CPRD-UK | Lincosamides | **3.522 [ 3.305 to 3.739]  p < 0.001** | 0.005 [-0.001 to 0.011]  p = 0.137 | 0.438 [-0.096 to 0.973]  p = 0.111 | **-0.054 [-0.091 to -0.018]  p = 0.004** | 0.428 [-0.071 to 0.927]  p = 0.096 | 0.027 [-0.012 to 0.066]  p = 0.182 |
| CPRD-UK | Macrolides | **432.697 [406.699 to 458.695]  p < 0.001** | **-2.533 [ -3.273 to -1.794]  p < 0.001** | 34.205 [-29.848 to 98.259]  p = 0.298 | -4.160 [ -8.521 to 0.200]  p = 0.064 | 36.609 [-23.214 to 96.431]  p = 0.233 | **7.546 [ 2.835 to 12.258]  p = 0.002** |
| CPRD-UK | Nitrofuran derivatives | **146.447 [136.863 to 156.030]  p < 0.001** | **2.168 [ 1.896 to 2.441]  p < 0.001** | 20.676 [ -2.935 to 44.288]  p = 0.089 | -0.630 [ -2.238 to 0.977]  p = 0.444 | -3.498 [-25.550 to 18.554]  p = 0.757 | **-2.499 [ -4.236 to -0.763]  p = 0.006** |
| CPRD-UK | Other antibacterials (J01XX) | -0.252 [-0.753 to 0.249]  p = 0.326 | **0.043 [ 0.029 to 0.057]  p < 0.001** | **1.821 [ 0.587 to 3.055]  p = 0.005** | **0.350 [ 0.266 to 0.434]  p < 0.001** | 0.690 [-0.463 to 1.842]  p = 0.244 | 0.032 [-0.059 to 0.123]  p = 0.490 |
| CPRD-UK | Other beta-lactam antibacterials | **75.556 [73.496 to 77.615]  p < 0.001** | **-0.512 [-0.571 to -0.454]  p < 0.001** | 3.683 [-1.392 to 8.757]  p = 0.158 | **0.772 [ 0.427 to 1.118]  p < 0.001** | 3.219 [-1.521 to 7.958]  p = 0.186 | **-0.513 [-0.886 to -0.140]  p = 0.008** |
| CPRD-UK | Penicillins with extended-spectrum | **1358.673 [1189.166 to 1528.180]  p < 0.001** | **-5.573 [ -10.394 to -0.752]  p = 0.026** | 208.957 [-208.676 to 626.590]  p = 0.329 | **-30.298 [ -58.731 to -1.866]  p = 0.039** | 301.595 [ -88.451 to 691.641]  p = 0.133 | **46.846 [ 16.126 to 77.565]  p = 0.004** |
| CPRD-UK | Sulfonamides and trimethoprim | **476.348 [463.558 to 489.137]  p < 0.001** | **-1.938 [ -2.302 to -1.575]  p < 0.001** | **-39.403 [-70.914 to -7.891]  p = 0.016** | **2.380 [ 0.234 to 4.525]  p = 0.032** | 5.871 [-23.559 to 35.301]  p = 0.697 | -0.999 [ -3.317 to 1.318]  p = 0.400 |
| CPRD-UK | Tetracyclines | **387.992 [347.570 to 428.414]  p < 0.001** | 0.756 [ -0.393 to 1.906]  p = 0.200 | 63.163 [-36.429 to 162.754]  p = 0.217 | **-7.371 [-14.151 to -0.590]  p = 0.036** | **94.184 [ 1.171 to 187.197]  p = 0.050** | **9.586 [ 2.260 to 16.912]  p = 0.012** |
| PHARMO-NL | Beta-lactamase resistant penicillins | **111.671 [ 99.518 to 123.824]  p < 0.001** | **0.803 [ 0.473 to 1.133]  p < 0.001** | -3.681 [-29.348 to 21.987]  p = 0.779 | -0.778 [ -2.801 to 1.246]  p = 0.453 | -28.993 [-63.141 to 5.155]  p = 0.100 | 3.064 [ -0.673 to 6.802]  p = 0.112 |
| PHARMO-NL | Beta-lactamase sensitive penicillins | **57.011 [ 51.620 to 62.403]  p < 0.001** | **-0.449 [ -0.596 to -0.303]  p < 0.001** | **22.655 [ 11.267 to 34.043]  p < 0.001** | **-1.335 [ -2.233 to -0.438]  p = 0.004** | -2.809 [-17.960 to 12.342]  p = 0.717 | **3.311 [ 1.653 to 4.969]  p < 0.001** |
| PHARMO-NL | Combinations of penicillins | **189.994 [179.508 to 200.481]  p < 0.001** | **0.766 [ 0.481 to 1.050]  p < 0.001** | -2.317 [-24.465 to 19.831]  p = 0.838 | **-3.498 [ -5.244 to -1.752]  p < 0.001** | -20.770 [-50.236 to 8.696]  p = 0.171 | **6.229 [ 3.004 to 9.454]  p < 0.001** |
| PHARMO-NL | Lincosamides | **14.043 [12.910 to 15.176]  p < 0.001** | **0.206 [ 0.175 to 0.237]  p < 0.001** | -0.508 [-2.901 to 1.885]  p = 0.678 | **-0.260 [-0.449 to -0.071]  p = 0.008** | 1.718 [-1.465 to 4.902]  p = 0.293 | 0.151 [-0.197 to 0.500]  p = 0.397 |
| PHARMO-NL | Macrolides | **148.943 [138.349 to 159.538]  p < 0.001** | **0.511 [ 0.223 to 0.798]  p = 0.001** | 4.200 [-18.176 to 26.577]  p = 0.714 | **-4.671 [ -6.435 to -2.907]  p < 0.001** | -23.084 [-52.853 to 6.686]  p = 0.132 | **8.183 [ 4.925 to 11.441]  p < 0.001** |
| PHARMO-NL | Nitrofuran derivatives | **247.625 [228.280 to 266.969]  p < 0.001** | **2.499 [ 1.974 to 3.025]  p < 0.001** | -18.255 [-59.112 to 22.602]  p = 0.384 | **-4.187 [ -7.408 to -0.966]  p = 0.013** | -36.087 [-90.443 to 18.269]  p = 0.197 | **8.317 [ 2.368 to 14.266]  p = 0.007** |
| PHARMO-NL | Other antibacterials (J01XX) | **8.781 [ 4.823 to 12.740]  p < 0.001** | **1.063 [ 0.956 to 1.171]  p < 0.001** | **-12.088 [-20.449 to -3.727]  p = 0.006** | **0.792 [ 0.133 to 1.451]  p = 0.021** | **-14.425 [-25.548 to -3.302]  p = 0.013** | **-1.786 [ -3.003 to -0.569]  p = 0.005** |
| PHARMO-NL | Other beta-lactam antibacterials | **2.417 [ 2.080 to 2.754]  p < 0.001** | 0.008 [-0.001 to 0.017]  p = 0.086 | 0.365 [-0.347 to 1.077]  p = 0.317 | 0.007 [-0.049 to 0.063]  p = 0.810 | -0.770 [-1.717 to 0.177]  p = 0.115 | 0.082 [-0.021 to 0.186]  p = 0.123 |
| PHARMO-NL | Penicillins with extended-spectrum | **398.914 [ 338.301 to 459.526]  p < 0.001** | **2.108 [ 0.461 to 3.754]  p = 0.014** | -36.657 [-164.675 to 91.360]  p = 0.576 | **-11.904 [ -21.995 to -1.813]  p = 0.023** | -63.648 [-233.962 to 106.666]  p = 0.466 | **23.237 [ 4.597 to 41.877]  p = 0.017** |
| PHARMO-NL | Sulfonamides and trimethoprim | **26.879 [ 24.820 to 28.939]  p < 0.001** | **0.230 [ 0.174 to 0.286]  p < 0.001** | -0.438 [ -4.788 to 3.911]  p = 0.844 | -0.313 [ -0.655 to 0.030]  p = 0.077 | -5.364 [-11.150 to 0.422]  p = 0.073 | **0.649 [ 0.016 to 1.282]  p = 0.048** |
| PHARMO-NL | Tetracyclines | **271.054 [ 235.426 to 306.681]  p < 0.001** | 0.146 [ -0.822 to 1.114]  p = 0.768 | -16.970 [ -92.217 to 58.277]  p = 0.660 | **-6.185 [ -12.116 to -0.253]  p = 0.044** | -45.383 [-145.492 to 54.725]  p = 0.377 | **11.275 [ 0.318 to 22.231]  p = 0.047** |

Drug classes with low cell counts (> 10% cells of monthly counts with values of 5 or less) were excluded.

# Covariate analyses for fluoroquinolone prescription patterns

All covariate analyses were conducted using a permissible gap of 30 days and excluding data during RMMs periods and 6-month lag periods.

## Interrupted time series regression results for fluoroquinolone prescription rates for incident use stratified by sex

| Database | Sex | (Intercept)  [95% CI] | Slope before RMMs  [95% CI] | Step change after 2018/19 RMMs [95% CI] | Slope change after 2018/19 RMMs  [95% CI] | Step change after 2020 RMMs  [95% CI] | Slope change after 2020 RMMs  [95% CI] |
| --- | --- | --- | --- | --- | --- | --- | --- |
| CPRD-UK | female | **56.338 [54.798 to 57.877]  p < 0.001** | **-0.105 [-0.149 to -0.062]  p < 0.001** | **-4.163 [-7.956 to -0.370]  p = 0.034** | **-0.768 [-1.026 to -0.510]  p < 0.001** | **4.550 [ 1.008 to 8.093]  p = 0.013** | **0.713 [ 0.434 to 0.992]  p < 0.001** |
| CPRD-UK | male | **70.728 [ 68.526 to 72.929]  p < 0.001** | -0.039 [ -0.102 to 0.023]  p = 0.222 | **-7.630 [-13.054 to -2.207]  p = 0.007** | **-0.592 [ -0.961 to -0.222]  p = 0.002** | 2.874 [ -2.191 to 7.939]  p = 0.269 | **0.416 [ 0.017 to 0.815]  p = 0.043** |
| PHARMO-NL | female | **107.829 [103.686 to 111.972]  p < 0.001** | -0.103 [ -0.221 to 0.015]  p = 0.090 | -6.453 [-17.561 to 4.654]  p = 0.258 | -0.703 [ -1.573 to 0.167]  p = 0.117 | -3.106 [-17.011 to 10.800]  p = 0.663 | **1.698 [ 0.213 to 3.183]  p = 0.028** |
| PHARMO-NL | male | **133.077 [128.584 to 137.571]  p < 0.001** | 0.111 [ -0.017 to 0.238]  p = 0.094 | -5.560 [-17.607 to 6.488]  p = 0.368 | **-0.962 [ -1.905 to -0.018]  p = 0.049** | -1.370 [-16.452 to 13.712]  p = 0.859 | **1.868 [ 0.258 to 3.478]  p = 0.026** |

## Interrupted time series regression results for fluoroquinolone prescription rates for incident use stratified by age group

| Database | Age group^A^ | (Intercept)  [95% CI] | Slope before RMMs  [95% CI] | Step change after 2018/19 RMMs [95% CI] | Slope change after 2018/19 RMMs  [95% CI] | Step change after 2020 RMMs  [95% CI] | Slope change after 2020 RMMs  [95% CI] |
| --- | --- | --- | --- | --- | --- | --- | --- |
| CPRD-UK | <2 years | 0.351 [-0.075 to 0.777]  p = 0.110 | 0.006 [-0.006 to 0.018]  p = 0.346 | -0.366 [-1.375 to 0.644]  p = 0.479 | 0.015 [-0.053 to 0.084]  p = 0.665 | -0.794 [-1.766 to 0.179]  p = 0.113 | -0.021 [-0.095 to 0.053]  p = 0.582 |
| CPRD-UK | 2 to <12 years | **3.328 [ 2.885 to 3.771]  p < 0.001** | **-0.024 [-0.037 to -0.012]  p < 0.001** | 0.976 [-0.116 to 2.068]  p = 0.083 | -0.037 [-0.111 to 0.037]  p = 0.332 | -0.486 [-1.481 to 0.510]  p = 0.341 | 0.075 [-0.005 to 0.155]  p = 0.069 |
| CPRD-UK | 12 to <19 years | **13.352 [12.029 to 14.675]  p < 0.001** | -0.022 [-0.060 to 0.016]  p = 0.252 | -0.542 [-3.802 to 2.718]  p = 0.745 | **-0.254 [-0.475 to -0.032]  p = 0.027** | 1.190 [-1.855 to 4.234]  p = 0.445 | **0.250 [ 0.010 to 0.489]  p = 0.044** |
| CPRD-UK | 19 to <30 years | **44.040 [41.961 to 46.119]  p < 0.001** | 0.031 [-0.028 to 0.090]  p = 0.304 | -4.469 [-9.592 to 0.653]  p = 0.090 | **-0.553 [-0.902 to -0.204]  p = 0.002** | 0.769 [-4.015 to 5.554]  p = 0.753 | 0.347 [-0.030 to 0.724]  p = 0.074 |
| CPRD-UK | 30 to <40 years | **49.080 [ 46.902 to 51.259]  p < 0.001** | 0.025 [ -0.037 to 0.087]  p = 0.428 | **-6.304 [-11.672 to -0.937]  p = 0.023** | **-0.475 [ -0.840 to -0.110]  p = 0.012** | **6.280 [ 1.267 to 11.293]  p = 0.016** | 0.174 [ -0.221 to 0.569]  p = 0.390 |
| CPRD-UK | 40 to <50 years | **65.386 [63.007 to 67.765]  p < 0.001** | **-0.142 [-0.209 to -0.074]  p < 0.001** | -3.285 [-9.147 to 2.576]  p = 0.275 | **-0.574 [-0.973 to -0.175]  p = 0.006** | 2.836 [-2.639 to 8.310]  p = 0.312 | **0.620 [ 0.189 to 1.051]  p = 0.006** |
| CPRD-UK | 50 to <60 years | **78.473 [ 75.759 to 81.186]  p < 0.001** | **-0.102 [ -0.179 to -0.025]  p = 0.011** | **-8.042 [-14.728 to -1.356]  p = 0.020** | **-0.650 [ -1.105 to -0.195]  p = 0.006** | 2.909 [ -3.335 to 9.153]  p = 0.363 | **0.556 [ 0.064 to 1.048]  p = 0.029** |
| CPRD-UK | 60 to <70 years | **102.184 [ 99.152 to 105.217]  p < 0.001** | **-0.156 [ -0.242 to -0.069]  p = 0.001** | **-9.070 [-16.542 to -1.598]  p = 0.019** | **-1.131 [ -1.639 to -0.622]  p < 0.001** | 5.852 [ -1.127 to 12.831]  p = 0.103 | **0.923 [ 0.373 to 1.473]  p = 0.001** |
| CPRD-UK | 70 to <80 years | **131.591 [127.287 to 135.896]  p < 0.001** | **-0.241 [ -0.363 to -0.118]  p < 0.001** | **-11.823 [-22.429 to -1.217]  p = 0.031** | **-1.554 [ -2.276 to -0.831]  p < 0.001** | 1.422 [ -8.484 to 11.327]  p = 0.779 | **1.619 [ 0.839 to 2.399]  p < 0.001** |
| CPRD-UK | 80 years and older | **151.063 [145.784 to 156.342]  p < 0.001** | **-0.227 [ -0.377 to -0.077]  p = 0.004** | **-23.581 [-36.588 to -10.573]  p = 0.001** | **-1.828 [ -2.713 to -0.942]  p < 0.001** | **13.262 [ 1.114 to 25.410]  p = 0.035** | **1.435 [ 0.478 to 2.392]  p = 0.004** |
| PHARMO-NL | <2 years | **0.787 [ 0.011 to 1.562]  p = 0.050** | -0.015 [-0.037 to 0.007]  p = 0.181 | 0.170 [-1.908 to 2.249]  p = 0.873 | 0.015 [-0.148 to 0.178]  p = 0.855 | 0.000 [-2.602 to 2.602]  p = 1.000 | 0.000 [-0.278 to 0.278]  p = 1.000 |
| PHARMO-NL | 2 to <12 years | **3.370 [ 2.621 to 4.119]  p < 0.001** | **-0.028 [-0.049 to -0.006]  p = 0.013** | -1.036 [-3.045 to 0.972]  p = 0.315 | **0.176 [ 0.019 to 0.333]  p = 0.031** | -0.234 [-2.748 to 2.280]  p = 0.856 | **-0.347 [-0.616 to -0.079]  p = 0.013** |
| PHARMO-NL | 12 to <19 years | **15.762 [13.275 to 18.249]  p < 0.001** | 0.014 [-0.056 to 0.085]  p = 0.693 | 1.662 [-5.006 to 8.331]  p = 0.626 | **-0.556 [-1.079 to -0.034]  p = 0.040** | 0.145 [-8.204 to 8.493]  p = 0.973 | **1.325 [ 0.433 to 2.216]  p = 0.005** |
| PHARMO-NL | 19 to <30 years | **52.383 [48.855 to 55.911]  p < 0.001** | 0.056 [-0.044 to 0.156]  p = 0.278 | -0.292 [-9.751 to 9.166]  p = 0.952 | -0.750 [-1.490 to -0.009]  p = 0.051 | 4.040 [-7.800 to 15.881]  p = 0.506 | 0.910 [-0.355 to 2.174]  p = 0.162 |
| PHARMO-NL | 30 to <40 years | **60.506 [56.575 to 64.438]  p < 0.001** | 0.002 [-0.110 to 0.113]  p = 0.977 | 1.333 [-9.207 to 11.874]  p = 0.805 | -0.741 [-1.566 to 0.085]  p = 0.082 | 7.809 [-5.386 to 21.005]  p = 0.250 | 0.372 [-1.036 to 1.781]  p = 0.606 |
| PHARMO-NL | 40 to <50 years | **83.017 [ 78.657 to 87.376]  p < 0.001** | -0.082 [ -0.206 to 0.042]  p = 0.199 | 3.586 [ -8.101 to 15.273]  p = 0.549 | **-1.155 [ -2.070 to -0.239]  p = 0.016** | 1.757 [-12.874 to 16.387]  p = 0.815 | 1.581 [ 0.019 to 3.143]  p = 0.051 |
| PHARMO-NL | 50 to <60 years | **129.551 [124.665 to 134.437]  p < 0.001** | **-0.278 [ -0.417 to -0.139]  p < 0.001** | 0.272 [-12.827 to 13.371]  p = 0.968 | **-1.453 [ -2.479 to -0.427]  p = 0.007** | 2.892 [-13.506 to 19.291]  p = 0.730 | **2.347 [ 0.596 to 4.098]  p = 0.010** |
| PHARMO-NL | 60 to <70 years | **217.260 [208.877 to 225.643]  p < 0.001** | **-0.386 [ -0.625 to -0.148]  p = 0.002** | -5.112 [-27.587 to 17.362]  p = 0.657 | **-2.424 [ -4.184 to -0.664]  p = 0.008** | 0.563 [-27.571 to 28.698]  p = 0.969 | **4.785 [ 1.781 to 7.789]  p = 0.003** |
| PHARMO-NL | 70 to <80 years | **337.173 [325.746 to 348.599]  p < 0.001** | **-0.674 [ -0.999 to -0.349]  p < 0.001** | -19.392 [-50.025 to 11.240]  p = 0.218 | -0.587 [ -2.986 to 1.813]  p = 0.633 | -15.673 [-54.021 to 22.675]  p = 0.425 | 3.124 [ -0.970 to 7.218]  p = 0.139 |
| PHARMO-NL | 80 years and older | **475.247 [ 455.523 to 494.971]  p < 0.001** | **-0.609 [ -1.170 to -0.048]  p = 0.036** | **-88.065 [-140.943 to -35.188]  p = 0.002** | 3.108 [ -1.033 to 7.249]  p = 0.145 | -43.222 [-109.418 to 22.973]  p = 0.204 | 1.759 [ -5.308 to 8.827]  p = 0.627 |

^A^Patients were assigned to age groups based on their age at the start of the treatment episode.

In the analysis, we used 10 age groups: <2 years, 2 to <12 years, 12 to <19 years, 19 to < 30 years, 30 to <40 years, 40 to <50 years, 50 to < 60 years, 60 to <70 years, 70 to <80 years, and 80 years and older.

## Interrupted time series regression results for fluoroquinolone prescription rates for incident use stratified by indication

| Type of use | Indication group | (Intercept)  [95% CI] | Slope before RMMs  [95% CI] | Step change after 2018/19 RMMs [95% CI] | Slope change after 2018/19 RMMs  [95% CI] | Step change after 2020 RMMs  [95% CI] | Slope change after 2020 RMMs  [95% CI] |
| --- | --- | --- | --- | --- | --- | --- | --- |
| CPRD-UK | Urinary tract infections | **3.025 [ 2.849 to 3.201]  p < 0.001** | 0.000 [-0.005 to 0.005]  p = 0.890 | -0.160 [-0.594 to 0.273]  p = 0.470 | **-0.100 [-0.129 to -0.070]  p < 0.001** | **0.536 [ 0.131 to 0.941]  p = 0.011** | **0.121 [ 0.089 to 0.153]  p < 0.001** |
| CPRD-UK | Other | **5.911 [ 5.641 to 6.181]  p < 0.001** | **-0.033 [-0.041 to -0.026]  p < 0.001** | 0.348 [-0.317 to 1.012]  p = 0.308 | **-0.085 [-0.130 to -0.040]  p < 0.001** | 0.339 [-0.282 to 0.960]  p = 0.287 | **0.121 [ 0.072 to 0.170]  p < 0.001** |
| CPRD-UK | Unknown | **54.801 [53.415 to 56.187]  p < 0.001** | -0.040 [-0.079 to 0.000]  p = 0.051 | **-6.022 [-9.436 to -2.607]  p = 0.001** | **-0.501 [-0.733 to -0.269]  p < 0.001** | 2.882 [-0.307 to 6.071]  p = 0.079 | **0.328 [ 0.077 to 0.580]  p = 0.012** |
| PHARMO-NL | Urinary tract infections | **15.363 [14.589 to 16.138]  p < 0.001** | 0.008 [-0.014 to 0.030]  p = 0.463 | **-2.197 [-4.274 to -0.120]  p = 0.041** | 0.041 [-0.122 to 0.203]  p = 0.625 | -2.019 [-4.619 to 0.581]  p = 0.132 | 0.022 [-0.255 to 0.300]  p = 0.875 |
| PHARMO-NL | Other | **12.298 [11.483 to 13.113]  p < 0.001** | **-0.027 [-0.050 to -0.004]  p = 0.024** | 0.313 [-1.872 to 2.498]  p = 0.779 | -0.117 [-0.288 to 0.054]  p = 0.183 | 0.539 [-2.196 to 3.274]  p = 0.700 | 0.265 [-0.027 to 0.557]  p = 0.079 |
| PHARMO-NL | Unknown | **93.496 [ 90.339 to 96.653]  p < 0.001** | 0.021 [ -0.068 to 0.111]  p = 0.642 | -4.595 [-13.059 to 3.870]  p = 0.291 | **-0.739 [ -1.402 to -0.076]  p = 0.032** | -1.111 [-11.707 to 9.485]  p = 0.838 | **1.520 [ 0.389 to 2.652]  p = 0.010** |

Indication groups other than urinary tract infections had low cell counts (> 10% of monthly aggregated cells with counts of 5 or less), so they were grouped in the analysis.

Indications initially were grouped as follows: upper respiratory tract infections, lower respiratory tract infections, urinary tract infections, ear infections, gastrointestinal infections (including hepatobiliary infections), genital infections (excluding testicular and prostatic infections, which were considered separately), testicular infections, prostatic infections, skin and soft tissue infections, bone infections, other indications (such as septicaemia, meningitis, infection of cerebrovascular fluid, and endocarditis), and unknown (indications either missing or not captured in the categories above).

## Interrupted time series regression results for fluoroquinolone prescription rates for incident use stratified by risk group

| Type of use | age group | (Intercept)  [95% CI] | Slope before RMMs  [95% CI] | Step change after 2018/19 RMMs [95% CI] | Slope change after 2018/19 RMMs  [95% CI] | Step change after 2020 RMMs  [95% CI] | Slope change after 2020 RMMs  [95% CI] |
| --- | --- | --- | --- | --- | --- | --- | --- |
| CPRD-UK | Risk for aortic aneurysm or dissection | **82.707 [ 80.662 to 84.751]  p < 0.001** | **-0.147 [ -0.205 to -0.088]  p < 0.001** | **-7.604 [-12.641 to -2.567]  p = 0.004** | **-0.933 [ -1.276 to -0.590]  p < 0.001** | **5.563 [ 0.858 to 10.267]  p = 0.022** | **0.819 [ 0.448 to 1.189]  p < 0.001** |
| CPRD-UK | Risk factors for tendinitis or tendon rupture | **44.752 [43.663 to 45.841]  p < 0.001** | **-0.081 [-0.112 to -0.050]  p < 0.001** | **-4.274 [-6.957 to -1.592]  p = 0.002** | **-0.507 [-0.690 to -0.325]  p < 0.001** | **2.935 [ 0.430 to 5.440]  p = 0.024** | **0.437 [ 0.239 to 0.634]  p < 0.001** |
| CPRD-UK | No risk factors | **18.527 [17.870 to 19.184]  p < 0.001** | 0.000 [-0.019 to 0.018]  p = 0.968 | **-2.005 [-3.624 to -0.386]  p = 0.017** | **-0.172 [-0.282 to -0.062]  p = 0.003** | 0.921 [-0.591 to 2.434]  p = 0.235 | **0.127 [ 0.008 to 0.246]  p = 0.039** |
| PHARMO-NL | Risk for aortic aneurysm or dissection | **57.981 [ 55.872 to 60.090]  p < 0.001** | **0.186 [ 0.126 to 0.246]  p < 0.001** | **-6.087 [-11.741 to -0.433]  p = 0.038** | -0.326 [ -0.769 to 0.116]  p = 0.152 | -4.954 [-12.032 to 2.124]  p = 0.174 | **0.886 [ 0.131 to 1.642]  p = 0.024** |
| PHARMO-NL | Risk factors for tendinitis or tendon rupture | **22.921 [21.878 to 23.965]  p < 0.001** | **0.155 [ 0.125 to 0.185]  p < 0.001** | -1.611 [-4.409 to 1.187]  p = 0.263 | **-0.233 [-0.452 to -0.014]  p = 0.041** | -2.451 [-5.954 to 1.052]  p = 0.174 | **0.607 [ 0.233 to 0.981]  p = 0.002** |
| PHARMO-NL | No risk factors | **55.634 [53.887 to 57.381]  p < 0.001** | **-0.186 [-0.235 to -0.136]  p < 0.001** | 0.093 [-4.590 to 4.776]  p = 0.969 | **-0.426 [-0.793 to -0.059]  p = 0.026** | 2.689 [-3.173 to 8.552]  p = 0.371 | **0.754 [ 0.128 to 1.380]  p = 0.021** |

Risk factors were grouped based on risk factors specified in Direct Healthcare Professional Communications: 1) Risk factors for aortic aneurysm or dissection: history of aortic aneurysm, aortic valve disorder, cerebrovascular disease, dyslipidaemia, dissection of aorta, hypertension, ischaemic heart disease, prior lipid-lowering medication use or prior tobacco use; 2) Risk factors for tendonitis or tendon rupture: concomitant glucocorticoid use, renal impairment, solid organ transplant, tendinitis, or prior tobacco use.

# Sensitivity analyses

## Prescription rates of any antiobiotics (ATC J01) and fluoroquinolones for incident use depending on definition of permissible gap in constructing treatment episodes.


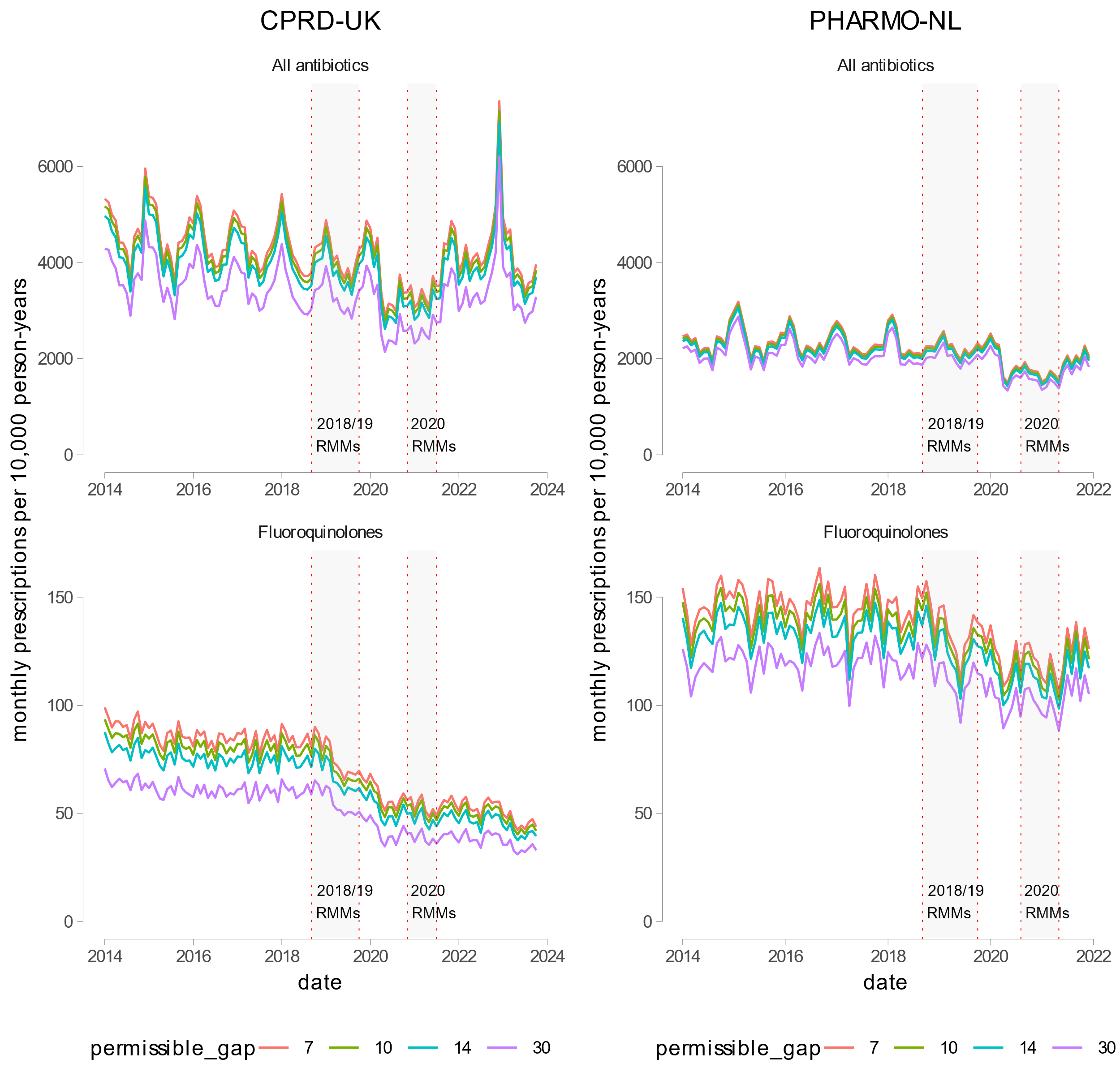


The left panel shows prescribing rates in CPRD-UK data, and the right panel shows fluoroquinolone prescribing rates in PHARMO-NL data. The top graphs show the prescription rates of incident use of any antibiotic, and the bottom graphs show prescription rates of incident use of fluoroquinolones depending on different permissible gap definitions in constructing treatment episodes. Lines indicate measured monthly prescriptions per 10,000 person-years (MPTPY) depending on the permissible gap used in the analysis (7, 10, 14, and 30). Vertical lines indicate risk minimization measures and their lag periods used in the analysis: 2018/19 RMMs – risk minimization measures from October 2018 until March 2019 (for both countries) with a six-month lag period (until September 2019). 2020 RMMs – risk minimization measures from September 2020 until October 2021 (for the Netherlands) or from December 2020 until June 2021 (for the UK) with six-month lag periods (May and June 2019, respectively). The high prescribing rates of penicillins with the extended spectrum, tetracyclines and macrolides in Decemeber 2022 seem to be related to the surge in type A streptococcus infections during that month [1].

[1] *Cunningham C, Fisher L, Wood C, et al. Incidence and treatment of group A streptococcal infections during covid-19 pandemic and 2022 outbreak: retrospective cohort study in England using OpenSAFELY-TPP. bmjmed 2024;* ***3****. doi:10.1136/bmjmed-2023-000791.*

## Interrupted time series regression analysis for fluoroquinolones depending on permissible gap length used in construction of treatment episodes and prescription type.

| Database | Prescription type | Permissible gap | (Intercept)  [95% CI] | Slope before RMMs  [95% CI] | Step change after 2018/19 RMMs [95% CI] | Slope change after 2018/19 RMMs  [95% CI] | Step change after 2020 RMMs  [95% CI] | Slope change after 2020 RMMs  [95% CI] |
| --- | --- | --- | --- | --- | --- | --- | --- | --- |
| CPRD-UK | incident | 7 | **90.653 [ 88.592 to 92.713]  p < 0.001** | **-0.148 [ -0.207 to -0.089]  p < 0.001** | **-8.472 [-13.548 to -3.395]  p = 0.001** | **-0.805 [ -1.151 to -0.459]  p < 0.001** | 4.130 [ -0.612 to 8.871]  p = 0.091 | **0.651 [ 0.277 to 1.024]  p = 0.001** |
| CPRD-UK | incident | 10 | **85.587 [ 83.619 to 87.555]  p < 0.001** | **-0.131 [ -0.187 to -0.075]  p < 0.001** | **-8.134 [-12.983 to -3.286]  p = 0.001** | **-0.781 [ -1.111 to -0.451]  p < 0.001** | 4.167 [ -0.361 to 8.695]  p = 0.074 | **0.622 [ 0.266 to 0.979]  p = 0.001** |
| CPRD-UK | incident | 14 | **79.374 [ 77.498 to 81.250]  p < 0.001** | **-0.113 [ -0.167 to -0.060]  p < 0.001** | **-7.756 [-12.379 to -3.133]  p = 0.001** | **-0.757 [ -1.072 to -0.442]  p < 0.001** | 4.210 [ -0.108 to 8.528]  p = 0.059 | **0.609 [ 0.269 to 0.949]  p = 0.001** |
| CPRD-UK | incident | 30 | **63.470 [61.860 to 65.081]  p < 0.001** | **-0.072 [-0.118 to -0.026]  p = 0.003** | **-5.887 [-9.855 to -1.920]  p = 0.004** | **-0.680 [-0.950 to -0.410]  p < 0.001** | 3.719 [ 0.014 to 7.425]  p = 0.052 | **0.564 [ 0.272 to 0.856]  p < 0.001** |
| CPRD-UK | add-on | 7 | **29.310 [28.319 to 30.301]  p < 0.001** | -0.017 [-0.045 to 0.012]  p = 0.253 | **-3.178 [-5.620 to -0.737]  p = 0.012** | **-0.305 [-0.471 to -0.139]  p < 0.001** | 0.368 [-1.913 to 2.648]  p = 0.753 | **0.263 [ 0.084 to 0.443]  p = 0.005** |
| CPRD-UK | add-on | 10 | **32.075 [30.966 to 33.184]  p < 0.001** | -0.018 [-0.050 to 0.013]  p = 0.263 | **-3.474 [-6.206 to -0.741]  p = 0.014** | **-0.336 [-0.522 to -0.150]  p = 0.001** | 0.415 [-2.137 to 2.967]  p = 0.751 | **0.291 [ 0.090 to 0.492]  p = 0.005** |
| CPRD-UK | add-on | 14 | **34.982 [33.688 to 36.277]  p < 0.001** | -0.016 [-0.052 to 0.021]  p = 0.406 | **-4.066 [-7.255 to -0.878]  p = 0.014** | **-0.367 [-0.584 to -0.150]  p = 0.001** | 0.600 [-2.378 to 3.577]  p = 0.694 | **0.309 [ 0.074 to 0.543]  p = 0.011** |
| CPRD-UK | add-on | 30 | **37.556 [ 35.870 to 39.242]  p < 0.001** | **0.049 [ 0.001 to 0.097]  p = 0.049** | **-6.602 [-10.755 to -2.448]  p = 0.002** | **-0.455 [ -0.738 to -0.172]  p = 0.002** | 0.526 [ -3.353 to 4.405]  p = 0.791 | **0.325 [ 0.020 to 0.631]  p = 0.039** |
| CPRD-UK | continued | 7 | **11.677 [11.224 to 12.130]  p < 0.001** | 0.003 [-0.010 to 0.016]  p = 0.684 | **-2.027 [-3.143 to -0.911]  p = 0.001** | -0.020 [-0.096 to 0.056]  p = 0.604 | -0.542 [-1.584 to 0.500]  p = 0.311 | -0.035 [-0.117 to 0.047]  p = 0.408 |
| CPRD-UK | continued | 10 | **12.998 [12.463 to 13.533]  p < 0.001** | 0.007 [-0.008 to 0.022]  p = 0.351 | **-2.430 [-3.747 to -1.113]  p < 0.001** | -0.029 [-0.118 to 0.061]  p = 0.534 | -0.640 [-1.870 to 0.590]  p = 0.310 | -0.040 [-0.137 to 0.057]  p = 0.424 |
| CPRD-UK | continued | 14 | **14.905 [14.252 to 15.559]  p < 0.001** | 0.015 [-0.003 to 0.034]  p = 0.106 | **-2.699 [-4.308 to -1.089]  p = 0.001** | -0.047 [-0.157 to 0.062]  p = 0.399 | -0.938 [-2.441 to 0.566]  p = 0.224 | -0.044 [-0.163 to 0.074]  p = 0.467 |
| CPRD-UK | continued | 30 | **18.771 [17.739 to 19.803]  p < 0.001** | **0.088 [ 0.059 to 0.117]  p < 0.001** | **-4.746 [-7.288 to -2.204]  p < 0.001** | **-0.198 [-0.371 to -0.025]  p = 0.027** | -0.340 [-2.714 to 2.034]  p = 0.780 | -0.032 [-0.219 to 0.155]  p = 0.736 |
| PHARMO-NL | incident | 7 | **147.512 [142.961 to 152.063]  p < 0.001** | -0.028 [ -0.157 to 0.101]  p = 0.672 | -9.289 [-21.489 to 2.911]  p = 0.140 | -0.905 [ -1.861 to 0.050]  p = 0.067 | -4.740 [-20.013 to 10.532]  p = 0.545 | **2.111 [ 0.480 to 3.741]  p = 0.013** |
| PHARMO-NL | incident | 10 | **141.620 [137.278 to 145.962]  p < 0.001** | -0.022 [ -0.146 to 0.101]  p = 0.727 | -9.309 [-20.949 to 2.331]  p = 0.121 | -0.837 [ -1.749 to 0.074]  p = 0.076 | -4.463 [-19.035 to 10.108]  p = 0.550 | **1.990 [ 0.434 to 3.545]  p = 0.014** |
| PHARMO-NL | incident | 14 | **134.805 [130.682 to 138.928]  p < 0.001** | -0.006 [ -0.123 to 0.111]  p = 0.920 | -8.315 [-19.368 to 2.738]  p = 0.144 | -0.868 [ -1.733 to -0.002]  p = 0.053 | -3.966 [-17.803 to 9.872]  p = 0.576 | **1.990 [ 0.513 to 3.468]  p = 0.010** |
| PHARMO-NL | incident | 30 | **120.136 [116.423 to 123.849]  p < 0.001** | 0.001 [ -0.104 to 0.107]  p = 0.983 | -6.048 [-16.002 to 3.906]  p = 0.237 | **-0.826 [ -1.606 to -0.047]  p = 0.041** | -2.183 [-14.644 to 10.278]  p = 0.732 | **1.778 [ 0.448 to 3.108]  p = 0.011** |
| PHARMO-NL | add-on | 7 | **31.853 [29.946 to 33.759]  p < 0.001** | **0.059 [ 0.005 to 0.113]  p = 0.037** | -2.716 [-7.827 to 2.394]  p = 0.301 | -0.260 [-0.660 to 0.140]  p = 0.207 | -3.574 [-9.972 to 2.823]  p = 0.277 | **0.811 [ 0.128 to 1.494]  p = 0.022** |
| PHARMO-NL | add-on | 10 | **35.968 [ 33.863 to 38.074]  p < 0.001** | **0.067 [ 0.007 to 0.127]  p = 0.032** | -2.973 [ -8.618 to 2.671]  p = 0.305 | -0.318 [ -0.760 to 0.124]  p = 0.163 | -3.964 [-11.030 to 3.103]  p = 0.275 | **0.915 [ 0.160 to 1.669]  p = 0.020** |
| PHARMO-NL | add-on | 14 | **40.219 [ 37.854 to 42.583]  p < 0.001** | **0.072 [ 0.005 to 0.139]  p = 0.039** | -4.212 [-10.551 to 2.127]  p = 0.197 | -0.297 [ -0.793 to 0.200]  p = 0.245 | -4.066 [-12.002 to 3.869]  p = 0.318 | **0.871 [ 0.024 to 1.719]  p = 0.047** |
| PHARMO-NL | add-on | 30 | **47.154 [ 44.118 to 50.190]  p < 0.001** | **0.120 [ 0.034 to 0.206]  p = 0.008** | -6.191 [-14.330 to 1.947]  p = 0.140 | -0.409 [ -1.046 to 0.229]  p = 0.213 | -4.817 [-15.006 to 5.371]  p = 0.357 | 1.000 [ -0.088 to 2.087]  p = 0.075 |
| PHARMO-NL | continued | 7 | **15.510 [14.471 to 16.549]  p < 0.001** | 0.007 [-0.023 to 0.036]  p = 0.664 | -2.774 [-5.560 to 0.012]  p = 0.055 | 0.056 [-0.162 to 0.274]  p = 0.618 | -0.550 [-4.038 to 2.937]  p = 0.758 | -0.002 [-0.375 to 0.370]  p = 0.991 |
| PHARMO-NL | continued | 10 | **16.553 [15.439 to 17.668]  p < 0.001** | 0.009 [-0.023 to 0.040]  p = 0.595 | -2.912 [-5.901 to 0.077]  p = 0.060 | 0.024 [-0.210 to 0.258]  p = 0.843 | -0.446 [-4.188 to 3.295]  p = 0.816 | 0.042 [-0.358 to 0.441]  p = 0.838 |
| PHARMO-NL | continued | 14 | **18.111 [16.837 to 19.385]  p < 0.001** | 0.008 [-0.028 to 0.045]  p = 0.655 | -3.055 [-6.471 to 0.360]  p = 0.083 | 0.013 [-0.254 to 0.281]  p = 0.922 | -0.814 [-5.090 to 3.462]  p = 0.710 | 0.074 [-0.382 to 0.531]  p = 0.751 |
| PHARMO-NL | continued | 30 | **21.935 [20.372 to 23.499]  p < 0.001** | 0.027 [-0.018 to 0.071]  p = 0.240 | **-4.652 [-8.844 to -0.461]  p = 0.033** | 0.022 [-0.307 to 0.350]  p = 0.897 | -1.771 [-7.018 to 3.476]  p = 0.510 | 0.157 [-0.403 to 0.717]  p = 0.585 |

## Interrupted time series regression analysis for incident use of fluoroquinolones considering different post-intervention lag periods.

| Database | Lag | (Intercept)  [95% CI] | Slope before RMMs  [95% CI] | Step change after 2018/19 RMMs [95% CI] | Slope change after 2018/19 RMMs  [95% CI] | Step change after 2020 RMMs  [95% CI] | Slope change after 2020 RMMs  [95% CI] |
| --- | --- | --- | --- | --- | --- | --- | --- |
| CPRD-UK | 0 | **63.763 [62.159 to 65.366]  p < 0.001** | **-0.087 [-0.135 to -0.039]  p = 0.001** | **-5.072 [-8.390 to -1.755]  p = 0.003** | **-0.706 [-0.943 to -0.469]  p < 0.001** | **3.871 [ 0.394 to 7.349]  p = 0.031** | **0.625 [ 0.371 to 0.879]  p < 0.001** |
| CPRD-UK | 1 | **63.763 [62.156 to 65.369]  p < 0.001** | **-0.087 [-0.135 to -0.039]  p = 0.001** | **-4.901 [-8.480 to -1.323]  p = 0.008** | **-0.718 [-0.973 to -0.463]  p < 0.001** | **4.430 [ 0.805 to 8.055]  p = 0.018** | **0.617 [ 0.343 to 0.891]  p < 0.001** |
| CPRD-UK | 2 | **63.763 [62.140 to 65.385]  p < 0.001** | **-0.087 [-0.136 to -0.038]  p = 0.001** | **-4.818 [-8.740 to -0.896]  p = 0.018** | **-0.724 [-1.003 to -0.445]  p < 0.001** | **4.522 [ 0.706 to 8.337]  p = 0.022** | **0.621 [ 0.323 to 0.919]  p < 0.001** |
| CPRD-UK | 3 | **63.763 [62.134 to 65.391]  p < 0.001** | **-0.087 [-0.136 to -0.038]  p = 0.001** | -4.181 [-8.489 to 0.127]  p = 0.060 | **-0.767 [-1.072 to -0.463]  p < 0.001** | **4.409 [ 0.407 to 8.411]  p = 0.033** | **0.680 [ 0.356 to 1.004]  p < 0.001** |
| CPRD-UK | 4 | **63.763 [62.126 to 65.400]  p < 0.001** | **-0.087 [-0.136 to -0.038]  p = 0.001** | -4.158 [-8.938 to 0.623]  p = 0.091 | **-0.769 [-1.103 to -0.435]  p < 0.001** | **4.904 [ 0.690 to 9.118]  p = 0.025** | **0.662 [ 0.307 to 1.017]  p < 0.001** |
| CPRD-UK | 5 | **63.763 [62.131 to 65.394]  p < 0.001** | **-0.087 [-0.136 to -0.038]  p = 0.001** | -4.412 [-9.719 to 0.896]  p = 0.107 | **-0.752 [-1.119 to -0.386]  p < 0.001** | **5.664 [ 1.253 to 10.075]  p = 0.014** | **0.611 [ 0.223 to 0.998]  p = 0.003** |
| CPRD-UK | 0 | **119.948 [116.124 to 123.773]  p < 0.001** | 0.010 [ -0.105 to 0.125]  p = 0.865 | **-9.780 [-18.208 to -1.353]  p = 0.026** | -0.685 [ -1.399 to 0.030]  p = 0.064 | -3.124 [-14.367 to 8.119]  p = 0.587 | **1.723 [ 0.544 to 2.902]  p = 0.005** |
| CPRD-UK | 1 | **119.948 [116.104 to 123.793]  p < 0.001** | 0.010 [ -0.105 to 0.125]  p = 0.866 | -9.256 [-18.542 to 0.030]  p = 0.054 | -0.728 [ -1.514 to 0.057]  p = 0.073 | -5.349 [-17.806 to 7.107]  p = 0.402 | **2.020 [ 0.704 to 3.335]  p = 0.003** |
| CPRD-UK | 2 | **119.948 [116.080 to 123.817]  p < 0.001** | 0.010 [ -0.106 to 0.126]  p = 0.867 | -7.661 [-18.016 to 2.693]  p = 0.151 | -0.858 [ -1.727 to 0.011]  p = 0.057 | -6.625 [-20.630 to 7.380]  p = 0.357 | **2.356 [ 0.875 to 3.837]  p = 0.003** |
| CPRD-UK | 3 | **119.948 [116.245 to 123.652]  p < 0.001** | 0.010 [ -0.101 to 0.121]  p = 0.861 | 0.051 [-11.059 to 11.160]  p = 0.993 | **-1.467 [ -2.388 to -0.545]  p = 0.003** | -3.367 [-18.581 to 11.848]  p = 0.666 | **3.021 [ 1.419 to 4.623]  p < 0.001** |
| CPRD-UK | 4 | **119.948 [116.229 to 123.668]  p < 0.001** | 0.010 [ -0.102 to 0.122]  p = 0.862 | 3.473 [ -9.183 to 16.129]  p = 0.592 | **-1.730 [ -2.763 to -0.697]  p = 0.002** | -0.676 [-18.326 to 16.974]  p = 0.940 | **3.183 [ 1.344 to 5.022]  p = 0.001** |
| PHARMO-NL | 5 | **119.948 [116.223 to 123.674]  p < 0.001** | 0.010 [ -0.102 to 0.122]  p = 0.862 | 6.633 [ -7.927 to 21.193]  p = 0.375 | **-1.967 [ -3.133 to -0.801]  p = 0.001** | -4.792 [-25.631 to 16.046]  p = 0.654 | **3.902 [ 1.767 to 6.036]  p = 0.001** |

Models estimated for add-on and continued use are available upon request.

## Prescription rates incident use of fluoroquinolones and 5 of the most frequently used antibiotics (ATC code J01) for incident use: analysis excluding data after the start of the COVID-19 pandemic and without considering post-intervention lag period.


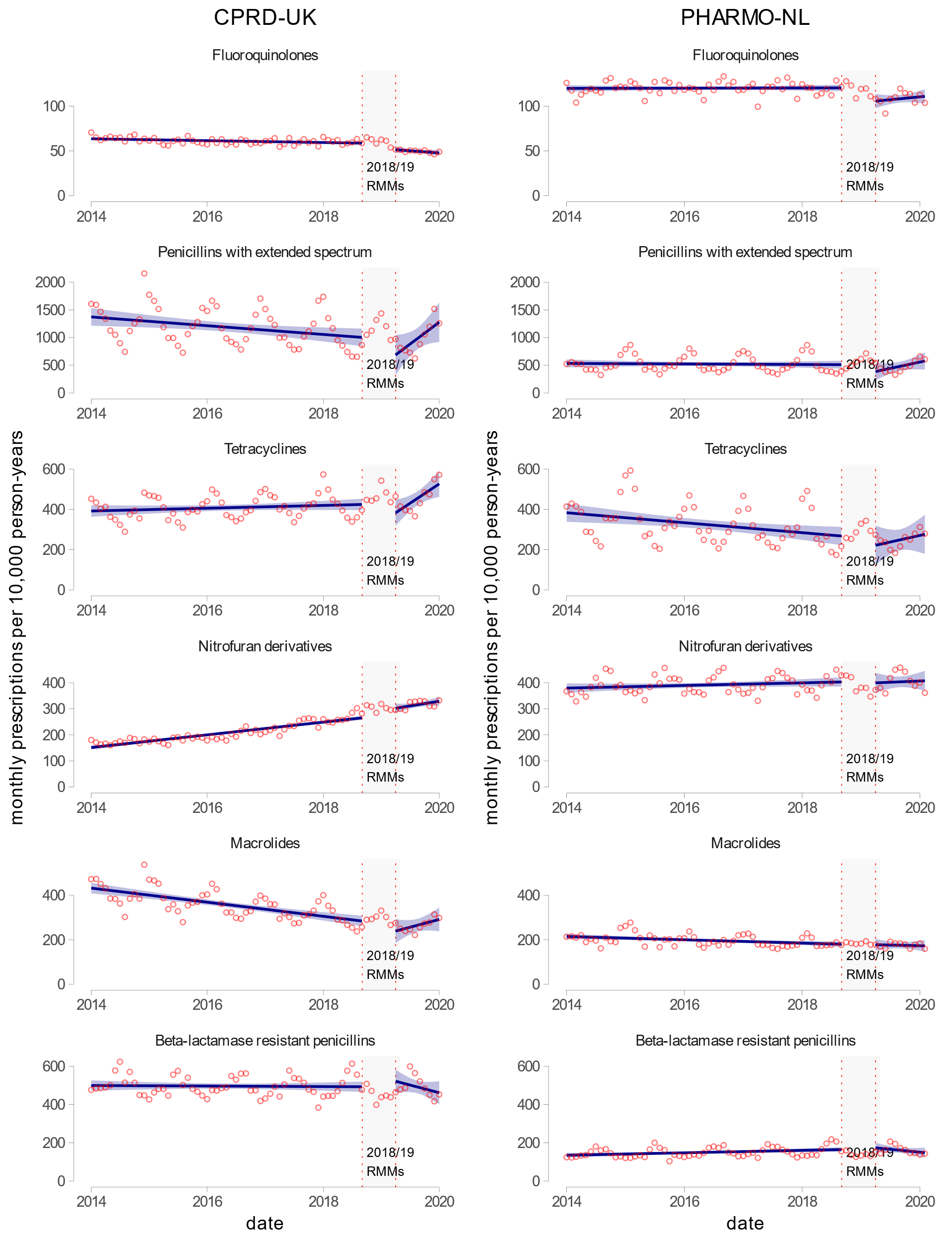


The left panel shows trends of fluoroquinolone use in CPRD-UK data (the UK), and the right panel shows fluoroquinolone prescribing trends in PHARMO-NL data(the Netherlands). Points in the graphs indicate measured monthly prescriptions, while lines indicate estimated prescribing rates based on interrupted time series (ITS) models. Rates are measured as monthly prescriptions per 10,000 person-years (MPTPY). Vertical lines indicate 2018/19 risk minimization measures – from October 2018 until March 2019 (for both countries) without considering a period. The lag period was not applied due to limited data points after the intervention and before the start of COVID-19 pandemic (4 for the UK and 5 for the Netherlands)

## Interrupted time series regression analysis for antibiotics (ATC code J01) incident use rates excluding the data after the start of the COVID-19 pandemic from the model estimation (and no lag window).

| Database | Drug class | (Intercept)  [95% CI] | Slope before RMMs  [95% CI] | Step change after 2018/19 RMMs [95% CI] | Slope change after 2018/19 RMMs  [95% CI] |
| --- | --- | --- | --- | --- | --- |
| CPRD-UK | Aminoglycosides | **0.227 [ 0.188 to 0.265]  p < 0.001** | **-0.002 [-0.003 to 0.000]  p = 0.012** | **0.112 [ 0.033 to 0.192]  p = 0.006** | -0.003 [-0.009 to 0.002]  p = 0.243 |
| CPRD-UK | Beta-lactamase resistant penicillins | **498.912 [472.220 to 525.604]  p < 0.001** | -0.113 [ -0.914 to 0.687]  p = 0.782 | 2.419 [-52.791 to 57.628]  p = 0.932 | -0.769 [ -4.707 to 3.169]  p = 0.703 |
| CPRD-UK | Beta-lactamase sensitive penicillins | **272.509 [227.516 to 317.503]  p < 0.001** | -0.335 [ -1.684 to 1.015]  p = 0.628 | 29.663 [-63.399 to 122.726]  p = 0.534 | -5.729 [-12.367 to 0.910]  p = 0.094 |
| CPRD-UK | Combinations of penicillins | **155.002 [150.758 to 159.245]  p < 0.001** | **-0.958 [ -1.085 to -0.830]  p < 0.001** | 6.360 [ -2.417 to 15.137]  p = 0.159 | 0.632 [ 0.006 to 1.258]  p = 0.051 |
| CPRD-UK | Fluoroquinolones | **63.763 [62.159 to 65.366]  p < 0.001** | **-0.087 [-0.135 to -0.039]  p = 0.001** | **-5.072 [-8.390 to -1.755]  p = 0.003** | **-0.706 [-0.943 to -0.469]  p < 0.001** |
| CPRD-UK | Lincosamides | **3.506 [ 3.281 to 3.730]  p < 0.001** | 0.005 [-0.001 to 0.012]  p = 0.114 | 0.353 [-0.112 to 0.818]  p = 0.139 | **-0.051 [-0.085 to -0.018]  p = 0.003** |
| CPRD-UK | Macrolides | **434.986 [408.264 to 461.709]  p < 0.001** | **-2.645 [ -3.446 to -1.843]  p < 0.001** | 20.315 [-34.957 to 75.587]  p = 0.473 | -2.951 [ -6.894 to 0.991]  p = 0.145 |
| CPRD-UK | Nitrofuran derivatives | **149.254 [139.697 to 158.811]  p < 0.001** | **2.029 [ 1.742 to 2.315]  p < 0.001** | **26.146 [ 6.380 to 45.913]  p = 0.011** | -0.551 [ -1.961 to 0.859]  p = 0.445 |
| CPRD-UK | Other antibacterials (J01XX) | -0.129 [-0.630 to 0.372]  p = 0.614 | **0.037 [ 0.022 to 0.052]  p < 0.001** | **1.982 [ 0.946 to 3.018]  p < 0.001** | **0.355 [ 0.281 to 0.429]  p < 0.001** |
| CPRD-UK | Other beta-lactam antibacterials | **75.922 [73.836 to 78.008]  p < 0.001** | **-0.531 [-0.593 to -0.468]  p < 0.001** | 4.025 [-0.290 to 8.340]  p = 0.070 | **0.803 [ 0.495 to 1.111]  p < 0.001** |
| CPRD-UK | Penicillins with extended spectrum | **1379.702 [1204.988 to 1554.416]  p < 0.001** | **-6.600 [ -11.841 to -1.360]  p = 0.015** | 110.243 [-251.129 to 471.616]  p = 0.551 | -20.808 [ -46.586 to 4.970]  p = 0.117 |
| CPRD-UK | Polymyxins | **0.949 [ 0.858 to 1.040]  p < 0.001** | **-0.003 [-0.006 to 0.000]  p = 0.031** | 0.175 [-0.013 to 0.363]  p = 0.072 | -0.005 [-0.018 to 0.009]  p = 0.487 |
| CPRD-UK | Sulfonamides and trimethoprim | **473.391 [460.625 to 486.157]  p < 0.001** | **-1.795 [ -2.177 to -1.412]  p < 0.001** | **-46.227 [-72.632 to -19.823]  p = 0.001** | **2.197 [ 0.313 to 4.080]  p = 0.024** |
| CPRD-UK | Tetracyclines | **391.651 [349.733 to 433.569]  p < 0.001** | 0.578 [ -0.680 to 1.835]  p = 0.370 | 42.877 [-43.825 to 129.579]  p = 0.335 | -5.741 [-11.926 to 0.444]  p = 0.072 |
| PHARMO-NL | Beta-lactamase resistant penicillins | **134.775 [122.017 to 147.533]  p < 0.001** | **0.530 [ 0.148 to 0.913]  p = 0.008** | -6.951 [-35.062 to 21.160]  p = 0.629 | -0.505 [ -2.888 to 1.879]  p = 0.679 |
| PHARMO-NL | Beta-lactamase sensitive penicillins | **76.255 [ 70.383 to 82.127]  p < 0.001** | **-0.849 [ -1.025 to -0.673]  p < 0.001** | **30.082 [ 17.144 to 43.021]  p < 0.001** | -0.893 [ -1.990 to 0.204]  p = 0.114 |
| PHARMO-NL | Combinations of penicillins | **262.172 [251.560 to 272.785]  p < 0.001** | **-0.495 [ -0.813 to -0.176]  p = 0.003** | 15.772 [ -7.610 to 39.155]  p = 0.190 | **-2.624 [ -4.606 to -0.641]  p = 0.011** |
| PHARMO-NL | Fluoroquinolones | **119.948 [116.124 to 123.773]  p < 0.001** | 0.010 [ -0.105 to 0.125]  p = 0.865 | **-9.780 [-18.208 to -1.353]  p = 0.026** | -0.685 [ -1.399 to 0.030]  p = 0.064 |
| PHARMO-NL | Lincosamides | **20.885 [19.723 to 22.047]  p < 0.001** | **0.094 [ 0.060 to 0.129]  p < 0.001** | 1.207 [-1.353 to 3.768]  p = 0.358 | **-0.222 [-0.439 to -0.005]  p = 0.049** |
| PHARMO-NL | Macrolides | **215.112 [203.107 to 227.116]  p < 0.001** | **-0.619 [ -0.979 to -0.259]  p = 0.001** | 23.117 [ -3.334 to 49.568]  p = 0.091 | **-4.276 [ -6.519 to -2.033]  p < 0.001** |
| PHARMO-NL | Nitrofuran derivatives | **378.732 [360.143 to 397.322]  p < 0.001** | 0.418 [ -0.140 to 0.975]  p = 0.146 | 9.643 [-31.318 to 50.604]  p = 0.646 | **-3.569 [ -7.043 to -0.096]  p = 0.047** |
| PHARMO-NL | Other antibacterials (J01XX) | **28.045 [ 23.699 to 32.391]  p < 0.001** | **0.930 [ 0.799 to 1.060]  p < 0.001** | **-12.327 [-21.902 to -2.751]  p = 0.014** | 0.505 [ -0.307 to 1.317]  p = 0.227 |
| PHARMO-NL | Other beta-lactam antibacterials | **3.677 [ 3.322 to 4.032]  p < 0.001** | **-0.013 [-0.023 to -0.002]  p = 0.023** | **0.884 [ 0.102 to 1.666]  p = 0.029** | -0.010 [-0.076 to 0.057]  p = 0.778 |
| PHARMO-NL | Penicillins with extended spectrum | **533.090 [ 463.220 to 602.959]  p < 0.001** | -0.400 [ -2.496 to 1.695]  p = 0.709 | 5.619 [-148.332 to 159.570]  p = 0.943 | -9.380 [ -22.435 to 3.676]  p = 0.163 |
| PHARMO-NL | Sulfonamides and trimethoprim | **53.211 [ 51.184 to 55.238]  p < 0.001** | **-0.186 [ -0.247 to -0.126]  p < 0.001** | **5.582 [ 1.116 to 10.048]  p = 0.016** | -0.072 [ -0.450 to 0.307]  p = 0.711 |
| PHARMO-NL | Tetracyclines | **385.699 [ 343.143 to 428.255]  p < 0.001** | **-2.064 [ -3.340 to -0.788]  p = 0.002** | 16.770 [ -76.997 to 110.538]  p = 0.727 | -3.885 [ -11.837 to 4.067]  p = 0.341 |

Drug classes with low cell counts (> 10% cells of monthly counts with values of 5 or less) were excluded. Models estimated for add-on and continued use are available upon request.

## Interrupted time series regression analysis for antibiotics (ATC code J01) incident use rates without exclusion of lag period from the model estimation

| Database | Drug class | (Intercept)  [95% CI] | Slope before RMMs  [95% CI] | Step change after 2018/19 RMMs [95% CI] | Slope change after 2018/19 RMMs  [95% CI] | Step change after 2020 RMMs  [95% CI] | Slope change after 2020 RMMs  [95% CI] |
| --- | --- | --- | --- | --- | --- | --- | --- |
| CPRD-UK | Beta-lactamase resistant penicillins | **498.912 [472.220 to 525.604]  p < 0.001** | -0.113 [ -0.914 to 0.687]  p = 0.782 | 2.419 [-52.791 to 57.628]  p = 0.932 | -0.769 [ -4.707 to 3.169]  p = 0.703 | 6.328 [-51.550 to 64.206]  p = 0.831 | 0.909 [ -3.320 to 5.139]  p = 0.674 |
| CPRD-UK | Beta-lactamase sensitive penicillins | **272.509 [227.516 to 317.503]  p < 0.001** | -0.335 [ -1.684 to 1.015]  p = 0.628 | 29.663 [-63.399 to 122.726]  p = 0.534 | -5.729 [-12.367 to 0.910]  p = 0.094 | 28.254 [-69.307 to 125.815]  p = 0.572 | **12.321 [ 5.191 to 19.451]  p = 0.001** |
| CPRD-UK | Combinations of penicillins | **155.002 [150.758 to 159.245]  p < 0.001** | **-0.958 [ -1.085 to -0.830]  p < 0.001** | 6.360 [ -2.417 to 15.137]  p = 0.159 | 0.632 [ 0.006 to 1.258]  p = 0.051 | 4.662 [ -4.540 to 13.863]  p = 0.323 | 0.604 [ -0.069 to 1.276]  p = 0.081 |
| CPRD-UK | Fluoroquinolones | **63.763 [62.159 to 65.366]  p < 0.001** | **-0.087 [-0.135 to -0.039]  p = 0.001** | **-5.072 [-8.390 to -1.755]  p = 0.003** | **-0.706 [-0.943 to -0.469]  p < 0.001** | **3.871 [ 0.394 to 7.349]  p = 0.031** | **0.625 [ 0.371 to 0.879]  p < 0.001** |
| CPRD-UK | Lincosamides | **3.506 [ 3.281 to 3.730]  p < 0.001** | 0.005 [-0.001 to 0.012]  p = 0.114 | 0.353 [-0.112 to 0.818]  p = 0.139 | **-0.051 [-0.085 to -0.018]  p = 0.003** | 0.306 [-0.182 to 0.793]  p = 0.222 | 0.027 [-0.008 to 0.063]  p = 0.137 |
| CPRD-UK | Macrolides | **434.986 [408.264 to 461.709]  p < 0.001** | **-2.645 [ -3.446 to -1.843]  p < 0.001** | 20.315 [-34.957 to 75.587]  p = 0.473 | -2.951 [ -6.894 to 0.991]  p = 0.145 | 23.479 [-34.464 to 81.423]  p = 0.429 | **6.809 [ 2.575 to 11.044]  p = 0.002** |
| CPRD-UK | Nitrofuran derivatives | **149.254 [139.697 to 158.811]  p < 0.001** | **2.029 [ 1.742 to 2.315]  p < 0.001** | **26.146 [ 6.380 to 45.913]  p = 0.011** | -0.551 [ -1.961 to 0.859]  p = 0.445 | -5.038 [-25.760 to 15.684]  p = 0.635 | **-2.306 [ -3.820 to -0.791]  p = 0.004** |
| CPRD-UK | Other antibacterials (J01XX) | -0.129 [-0.630 to 0.372]  p = 0.614 | **0.037 [ 0.022 to 0.052]  p < 0.001** | **1.982 [ 0.946 to 3.018]  p < 0.001** | **0.355 [ 0.281 to 0.429]  p < 0.001** | 0.738 [-0.348 to 1.824]  p = 0.186 | 0.037 [-0.043 to 0.116]  p = 0.369 |
| CPRD-UK | Other beta-lactam antibacterials | **75.922 [73.836 to 78.008]  p < 0.001** | **-0.531 [-0.593 to -0.468]  p < 0.001** | 4.025 [-0.290 to 8.340]  p = 0.070 | **0.803 [ 0.495 to 1.111]  p < 0.001** | 2.974 [-1.550 to 7.497]  p = 0.200 | **-0.509 [-0.839 to -0.178]  p = 0.003** |
| CPRD-UK | Penicillins with extended spectrum | **1379.702 [1204.988 to 1554.416]  p < 0.001** | **-6.600 [ -11.841 to -1.360]  p = 0.015** | 110.243 [-251.129 to 471.616]  p = 0.551 | -20.808 [ -46.586 to 4.970]  p = 0.117 | 181.141 [-197.700 to 559.981]  p = 0.351 | **41.875 [ 14.189 to 69.561]  p = 0.004** |
| CPRD-UK | Sulfonamides and trimethoprim | **473.391 [460.625 to 486.157]  p < 0.001** | **-1.795 [ -2.177 to -1.412]  p < 0.001** | **-46.227 [-72.632 to -19.823]  p = 0.001** | **2.197 [ 0.313 to 4.080]  p = 0.024** | 3.131 [-24.550 to 30.812]  p = 0.825 | -0.784 [ -2.807 to 1.239]  p = 0.449 |
| CPRD-UK | Tetracyclines | **391.651 [349.733 to 433.569]  p < 0.001** | 0.578 [ -0.680 to 1.835]  p = 0.370 | 42.877 [-43.825 to 129.579]  p = 0.335 | -5.741 [-11.926 to 0.444]  p = 0.072 | 66.506 [-24.387 to 157.399]  p = 0.155 | **9.160 [ 2.517 to 15.802]  p = 0.008** |
| PHARMO-NL | Beta-lactamase resistant penicillins | **134.775 [122.017 to 147.533]  p < 0.001** | **0.530 [ 0.148 to 0.913]  p = 0.008** | -6.951 [-35.062 to 21.160]  p = 0.629 | -0.505 [ -2.888 to 1.879]  p = 0.679 | -29.628 [-67.130 to 7.875]  p = 0.125 | 3.049 [ -0.884 to 6.982]  p = 0.132 |
| PHARMO-NL | Beta-lactamase sensitive penicillins | **76.255 [ 70.383 to 82.127]  p < 0.001** | **-0.849 [ -1.025 to -0.673]  p < 0.001** | **30.082 [ 17.144 to 43.021]  p < 0.001** | -0.893 [ -1.990 to 0.204]  p = 0.114 | -3.319 [-20.580 to 13.942]  p = 0.707 | **3.229 [ 1.419 to 5.039]  p = 0.001** |
| PHARMO-NL | Combinations of penicillins | **262.172 [251.560 to 272.785]  p < 0.001** | **-0.495 [ -0.813 to -0.176]  p = 0.003** | 15.772 [ -7.610 to 39.155]  p = 0.190 | **-2.624 [ -4.606 to -0.641]  p = 0.011** | -19.988 [-51.182 to 11.206]  p = 0.213 | **6.640 [ 3.369 to 9.911]  p < 0.001** |
| PHARMO-NL | Fluoroquinolones | **119.948 [116.124 to 123.773]  p < 0.001** | 0.010 [ -0.105 to 0.125]  p = 0.865 | **-9.780 [-18.208 to -1.353]  p = 0.026** | -0.685 [ -1.399 to 0.030]  p = 0.064 | -3.124 [-14.367 to 8.119]  p = 0.587 | **1.723 [ 0.544 to 2.902]  p = 0.005** |
| PHARMO-NL | Lincosamides | **20.885 [19.723 to 22.047]  p < 0.001** | **0.094 [ 0.060 to 0.129]  p < 0.001** | 1.207 [-1.353 to 3.768]  p = 0.358 | **-0.222 [-0.439 to -0.005]  p = 0.049** | 2.310 [-1.106 to 5.726]  p = 0.189 | 0.224 [-0.134 to 0.582]  p = 0.223 |
| PHARMO-NL | Macrolides | **215.112 [203.107 to 227.116]  p < 0.001** | **-0.619 [ -0.979 to -0.259]  p = 0.001** | 23.117 [ -3.334 to 49.568]  p = 0.091 | **-4.276 [ -6.519 to -2.033]  p < 0.001** | -18.352 [-53.640 to 16.936]  p = 0.311 | **8.964 [ 5.264 to 12.665]  p < 0.001** |
| PHARMO-NL | Nitrofuran derivatives | **378.732 [360.143 to 397.322]  p < 0.001** | 0.418 [ -0.140 to 0.975]  p = 0.146 | 9.643 [-31.318 to 50.604]  p = 0.646 | **-3.569 [ -7.043 to -0.096]  p = 0.047** | -23.062 [-77.707 to 31.583]  p = 0.411 | **9.708 [ 3.977 to 15.438]  p = 0.001** |
| PHARMO-NL | Other antibacterials (J01XX) | **28.045 [ 23.699 to 32.391]  p < 0.001** | **0.930 [ 0.799 to 1.060]  p < 0.001** | **-12.327 [-21.902 to -2.751]  p = 0.014** | 0.505 [ -0.307 to 1.317]  p = 0.227 | -10.241 [-23.016 to 2.534]  p = 0.120 | **-1.526 [ -2.866 to -0.186]  p = 0.028** |
| PHARMO-NL | Other beta-lactam antibacterials | **3.677 [ 3.322 to 4.032]  p < 0.001** | **-0.013 [-0.023 to -0.002]  p = 0.023** | **0.884 [ 0.102 to 1.666]  p = 0.029** | -0.010 [-0.076 to 0.057]  p = 0.778 | -0.455 [-1.498 to 0.589]  p = 0.396 | **0.135 [ 0.025 to 0.244]  p = 0.018** |
| PHARMO-NL | Penicillins with extended spectrum | **533.090 [ 463.220 to 602.959]  p < 0.001** | -0.400 [ -2.496 to 1.695]  p = 0.709 | 5.619 [-148.332 to 159.570]  p = 0.943 | -9.380 [ -22.435 to 3.676]  p = 0.163 | -73.512 [-278.893 to 131.870]  p = 0.485 | **23.370 [ 1.832 to 44.907]  p = 0.036** |
| PHARMO-NL | Sulfonamides and trimethoprim | **53.211 [ 51.184 to 55.238]  p < 0.001** | **-0.186 [ -0.247 to -0.126]  p < 0.001** | **5.582 [ 1.116 to 10.048]  p = 0.016** | -0.072 [ -0.450 to 0.307]  p = 0.711 | -4.530 [-10.488 to 1.428]  p = 0.140 | **0.808 [ 0.184 to 1.433]  p = 0.013** |
| PHARMO-NL | Tetracyclines | **385.699 [ 343.143 to 428.255]  p < 0.001** | **-2.064 [ -3.340 to -0.788]  p = 0.002** | 16.770 [ -76.997 to 110.538]  p = 0.727 | -3.885 [ -11.837 to 4.067]  p = 0.341 | -50.734 [-175.826 to 74.359]  p = 0.429 | 11.141 [ -1.976 to 24.259]  p = 0.100 |

Drug classes with low cell counts (> 10% cells of monthly counts with values of 5 or less) were excluded. Models estimated for add-on and continued use are available upon request.

## Interrupted time series regression analysis for antibiotics (ATC coode J01) incident use rates with inclusion of seasonal trends

| Database | Drug class | (Intercept)  [95% CI] | Slope before RMMs  [95% CI] | Step change after 2018/19 RMMs [95% CI] | Slope change after 2018/19 RMMs  [95% CI] | Step change after 2020 RMMs  [95% CI] | Slope change after 2020 RMMs  [95% CI] |
| --- | --- | --- | --- | --- | --- | --- | --- |
| CPRD-UK | Beta-lactamase resistant penicillins | **448.359 [427.923 to 468.794]  p < 0.001** | -0.392 [ -0.797 to 0.014]  p = 0.062 | 6.497 [-21.569 to 34.563]  p = 0.651 | -0.600 [ -2.613 to 1.414]  p = 0.561 | 25.975 [ -3.767 to 55.718]  p = 0.090 | 0.288 [ -1.869 to 2.444]  p = 0.794 |
| CPRD-UK | Beta-lactamase sensitive penicillins | **286.337 [ 229.644 to 343.029]  p < 0.001** | -0.080 [ -1.206 to 1.047]  p = 0.890 | 31.786 [ -46.075 to 109.646]  p = 0.426 | **-5.934 [ -11.520 to -0.347]  p = 0.040** | 2.514 [ -79.997 to 85.025]  p = 0.953 | **13.189 [ 7.206 to 19.171]  p < 0.001** |
| CPRD-UK | Combinations of penicillins | **155.769 [149.550 to 161.988]  p < 0.001** | **-0.951 [ -1.074 to -0.827]  p < 0.001** | 7.293 [ -1.248 to 15.834]  p = 0.098 | 0.573 [ -0.040 to 1.186]  p = 0.070 | 4.129 [ -4.922 to 13.181]  p = 0.374 | **0.686 [ 0.030 to 1.342]  p = 0.043** |
| CPRD-UK | Fluoroquinolones | **64.439 [62.222 to 66.655]  p < 0.001** | **-0.086 [-0.130 to -0.042]  p < 0.001** | **-4.160 [-7.205 to -1.116]  p = 0.009** | **-0.790 [-1.009 to -0.572]  p < 0.001** | **4.718 [ 1.492 to 7.944]  p = 0.005** | **0.703 [ 0.469 to 0.937]  p < 0.001** |
| CPRD-UK | Lincosamides | **3.284 [ 2.951 to 3.617]  p < 0.001** | 0.005 [-0.002 to 0.011]  p = 0.173 | 0.353 [-0.105 to 0.810]  p = 0.134 | **-0.050 [-0.083 to -0.017]  p = 0.003** | 0.369 [-0.116 to 0.853]  p = 0.139 | 0.024 [-0.011 to 0.059]  p = 0.178 |
| CPRD-UK | Macrolides | **464.852 [ 437.535 to 492.169]  p < 0.001** | **-2.474 [ -3.016 to -1.931]  p < 0.001** | 27.279 [ -10.238 to 64.795]  p = 0.157 | **-3.637 [ -6.329 to -0.945]  p = 0.009** | 14.109 [ -25.648 to 53.867]  p = 0.488 | **7.822 [ 4.939 to 10.705]  p < 0.001** |
| CPRD-UK | Nitrofuran derivatives | **147.119 [135.340 to 158.898]  p < 0.001** | **1.977 [ 1.743 to 2.211]  p < 0.001** | **33.450 [ 17.273 to 49.628]  p < 0.001** | -1.117 [ -2.278 to 0.043]  p = 0.062 | 5.696 [-11.448 to 22.840]  p = 0.516 | **-1.882 [ -3.126 to -0.639]  p = 0.004** |
| CPRD-UK | Other antibacterials (J01XX) | -0.492 [-1.234 to 0.250]  p = 0.197 | **0.036 [ 0.021 to 0.051]  p < 0.001** | **2.182 [ 1.162 to 3.201]  p < 0.001** | **0.340 [ 0.267 to 0.413]  p < 0.001** | 1.007 [-0.074 to 2.087]  p = 0.071 | 0.048 [-0.030 to 0.127]  p = 0.228 |
| CPRD-UK | Other beta-lactam antibacterials | **77.947 [74.885 to 81.008]  p < 0.001** | **-0.526 [-0.587 to -0.465]  p < 0.001** | **4.664 [ 0.460 to 8.868]  p = 0.032** | **0.749 [ 0.447 to 1.051]  p < 0.001** | 2.986 [-1.469 to 7.442]  p = 0.192 | **-0.443 [-0.766 to -0.120]  p = 0.009** |
| CPRD-UK | Penicillins with extended spectrum | **1627.660 [1481.836 to 1773.484]  p < 0.001** | **-5.391 [ -8.287 to -2.494]  p < 0.001** | 162.673 [ -37.599 to 362.945]  p = 0.115 | **-26.568 [ -40.937 to -12.198]  p < 0.001** | 136.880 [ -75.354 to 349.114]  p = 0.209 | **49.623 [ 34.234 to 65.011]  p < 0.001** |
| CPRD-UK | Polymyxins | **1.049 [ 0.912 to 1.185]  p < 0.001** | **-0.003 [-0.006 to -0.001]  p = 0.022** | 0.176 [-0.012 to 0.363]  p = 0.069 | -0.004 [-0.018 to 0.009]  p = 0.553 | **0.345 [ 0.146 to 0.544]  p = 0.001** | -0.004 [-0.019 to 0.010]  p = 0.563 |
| CPRD-UK | Sulfonamides and trimethoprim | **471.974 [456.499 to 487.449]  p < 0.001** | **-1.843 [ -2.150 to -1.535]  p < 0.001** | **-35.564 [-56.818 to -14.311]  p = 0.001** | 1.283 [ -0.242 to 2.808]  p = 0.102 | 18.095 [ -4.427 to 40.618]  p = 0.119 | -0.041 [ -1.674 to 1.592]  p = 0.960 |
| CPRD-UK | Tetracyclines | **452.835 [ 408.263 to 497.406]  p < 0.001** | 0.872 [ -0.013 to 1.758]  p = 0.056 | 52.603 [ -8.611 to 113.817]  p = 0.095 | **-6.874 [ -11.266 to -2.481]  p = 0.003** | 52.145 [ -12.725 to 117.016]  p = 0.119 | **10.792 [ 6.088 to 15.495]  p < 0.001** |
| PHARMO-NL | Beta-lactamase resistant penicillins | **116.714 [106.247 to 127.182]  p < 0.001** | **0.387 [ 0.194 to 0.580]  p < 0.001** | -1.651 [-15.833 to 12.530]  p = 0.820 | -0.785 [ -1.992 to 0.423]  p = 0.207 | -6.717 [-25.788 to 12.354]  p = 0.492 | 1.770 [ -0.253 to 3.793]  p = 0.091 |
| PHARMO-NL | Beta-lactamase sensitive penicillins | **80.856 [ 71.823 to 89.888]  p < 0.001** | **-0.812 [ -0.978 to -0.645]  p < 0.001** | **30.377 [ 18.140 to 42.615]  p < 0.001** | -0.974 [ -2.016 to 0.068]  p = 0.071 | -8.400 [-24.856 to 8.056]  p = 0.320 | **3.808 [ 2.063 to 5.554]  p < 0.001** |
| PHARMO-NL | Combinations of penicillins | **281.771 [269.157 to 294.385]  p < 0.001** | **-0.418 [ -0.650 to -0.185]  p = 0.001** | **20.446 [ 3.357 to 37.535]  p = 0.022** | **-3.113 [ -4.567 to -1.658]  p < 0.001** | **-29.023 [-52.004 to -6.042]  p = 0.016** | **8.277 [ 5.840 to 10.715]  p < 0.001** |
| PHARMO-NL | Fluoroquinolones | **121.936 [116.873 to 127.000]  p < 0.001** | 0.000 [ -0.093 to 0.093]  p = 0.999 | **-8.231 [-15.090 to -1.371]  p = 0.021** | **-0.717 [ -1.301 to -0.133]  p = 0.019** | -1.793 [-11.018 to 7.432]  p = 0.704 | **1.495 [ 0.516 to 2.473]  p = 0.004** |
| PHARMO-NL | Lincosamides | **20.839 [18.953 to 22.726]  p < 0.001** | **0.094 [ 0.060 to 0.129]  p < 0.001** | 1.081 [-1.474 to 3.637]  p = 0.410 | -0.201 [-0.418 to 0.017]  p = 0.075 | 2.114 [-1.323 to 5.550]  p = 0.232 | 0.185 [-0.179 to 0.550]  p = 0.323 |
| PHARMO-NL | Macrolides | **233.642 [218.284 to 249.000]  p < 0.001** | **-0.543 [ -0.826 to -0.260]  p < 0.001** | **28.561 [ 7.755 to 49.368]  p = 0.009** | **-4.820 [ -6.591 to -3.049]  p < 0.001** | -26.910 [-54.891 to 1.070]  p = 0.064 | **10.627 [ 7.658 to 13.595]  p < 0.001** |
| PHARMO-NL | Nitrofuran derivatives | **364.804 [347.500 to 382.109]  p < 0.001** | 0.222 [ -0.097 to 0.541]  p = 0.177 | 18.052 [ -5.391 to 41.496]  p = 0.136 | **-3.519 [ -5.515 to -1.523]  p = 0.001** | 0.330 [-31.197 to 31.856]  p = 0.984 | **6.714 [ 3.370 to 10.058]  p < 0.001** |
| PHARMO-NL | Other antibacterials (J01XX) | **22.546 [ 16.965 to 28.126]  p < 0.001** | **0.889 [ 0.786 to 0.992]  p < 0.001** | **-11.590 [-19.150 to -4.029]  p = 0.004** | 0.573 [ -0.071 to 1.217]  p = 0.085 | -5.211 [-15.378 to 4.956]  p = 0.319 | **-2.229 [ -3.308 to -1.151]  p < 0.001** |
| PHARMO-NL | Other beta-lactam antibacterials | **3.738 [ 3.148 to 4.328]  p < 0.001** | **-0.013 [-0.024 to -0.002]  p = 0.022** | **0.974 [ 0.175 to 1.773]  p = 0.020** | -0.016 [-0.084 to 0.052]  p = 0.648 | -0.366 [-1.440 to 0.709]  p = 0.507 | **0.134 [ 0.020 to 0.248]  p = 0.025** |
| PHARMO-NL | Penicillins with extended spectrum | **673.220 [ 612.684 to 733.756]  p < 0.001** | 0.422 [ -0.694 to 1.538]  p = 0.461 | 20.302 [ -61.710 to 102.315]  p = 0.629 | **-12.021 [ -19.003 to -5.039]  p = 0.001** | **-181.136 [-291.424 to -70.847]  p = 0.002** | **37.239 [ 25.540 to 48.938]  p < 0.001** |
| PHARMO-NL | Sulfonamides and trimethoprim | **52.690 [49.728 to 55.653]  p < 0.001** | **-0.194 [-0.249 to -0.140]  p < 0.001** | **6.001 [ 1.988 to 10.014]  p = 0.005** | -0.051 [-0.393 to 0.291]  p = 0.770 | -4.044 [-9.441 to 1.353]  p = 0.146 | **0.633 [ 0.060 to 1.205]  p = 0.034** |
| PHARMO-NL | Tetracyclines | **479.537 [ 438.264 to 520.810]  p < 0.001** | **-1.578 [ -2.339 to -0.818]  p < 0.001** | 24.143 [ -31.772 to 80.059]  p = 0.400 | **-5.204 [ -9.965 to -0.444]  p = 0.036** | **-112.619 [-187.813 to -37.425]  p = 0.004** | **18.567 [ 10.591 to 26.543]  p < 0.001** |

Drug classes with low cell counts (> 10% cells of monthly counts with values of 5 or less) were excluded. Models estimated for add-on and continued use are available upon request.
